# Low molecular weight organic acids stabilise siderite against oxidation and influence the composition of iron (oxyhydr)oxide oxidation products†

**DOI:** 10.1039/d4em00363b

**Published:** 2024-11-25

**Authors:** Katherine A. Rothwell, Laurel K. ThomasArrigo, Ralf Kaegi, Ruben Kretzschmar

**Affiliations:** a School of Earth Sciences Wills Memorial Building Bristol BS8 1RJ UK k.rothwell@bristol.ac.uk; b Environmental Chemistry Group, Institute of Chemistry, University of Neuchâtel Avenue de Bellevaux 51 2000 Neuchâtel Switzerland; c EAWAG, Swiss Federal Institute of Aquatic Science and Technology Überlandstrasse 133 8600 Dübendorf Switzerland; d Soil Chemistry Group, Institute of Biogeochemistry and Pollutant Dynamics, ETH Zurich, CHN Universitätstrasse 16 8092 Zurich Switzerland

## Abstract

Siderite (FeCO_3_) is an important reservoir of mineral-bound ferrous iron in non-sulfidic, reducing soils and sediments. It is redox sensitive, and its oxidation may facilitate the reduction of a range of pollutants, produce reactive oxygen species, or induce the formation of oxidation products with large surface areas for contaminant sorption. However, there is currently a limited understanding of the stability of siderite in complex environments such as soils and sediments. Here, we use a series of batch experiments complemented with thorough characterisation of mineral oxidation products to investigate the oxidation of siderite in the presence and absence of the low molecular weight organic acids (LMWOAs) citrate, tiron, salicylate, and EDTA as analogues for naturally occurring compounds or functional groups of natural organic matter that ubiquitously coexist with siderite. Our results show that siderite alone at pH 7.5 was completely oxidised to form ferrihydrite, nanocrystalline lepidocrocite, and nanocrystalline goethite in less than 6 hours. However, in the presence of LMWOAs, up to 48% of the siderite was preserved for more than 500 hours and the formation of goethite was inhibited in favour of ferrihydrite and lepidocrocite. Using experimental data from electron microscopy and chemical speciation modelling, we hypothesise that the siderite may be preserved through the formation of an Fe(iii)-passivation layer at the siderite surface.

## Abstract 2

Siderite is stabilised against oxidation in the presence of Low Molecular Weight Organic Acids (LMWOAs) with implications for iron mineral transformations and therefore contaminant and nutrient cycling.

## Graphical abstract

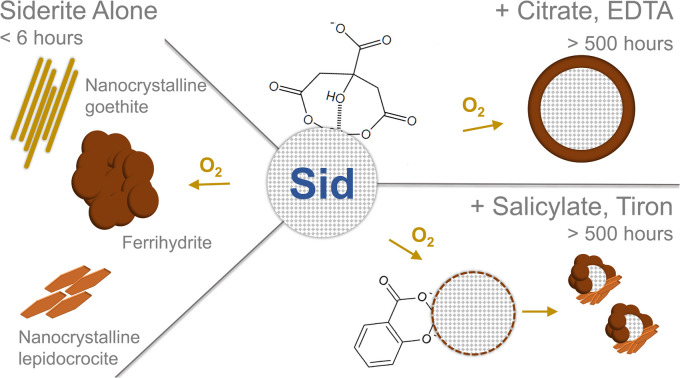

**Environmental significance**

The oxidation of siderite can be coupled to the reduction of various environmental pollutants, produce reactive oxygen species, or form products with large surface areas for contaminant sorption. However, our data shows that in the presence of low molecular weight organic acids (LMWOAs) siderite is considerably more stable against oxidation than previously thought. Thus, in complex biogeochemical environments such as periodically reducing soils and sediments, where both siderite and organic matter are likely ubiquitous, siderite is likely to persist for longer than previously anticipated under oxic conditions, with implications for its reactivity and therefore for the cycles of redox sensitive contaminants and nutrients.

## 1 Introduction

The biogeochemical cycle of iron is fundamentally important in natural systems^1–5^ and influences systems and processes ranging from the carbon cycle^6^ to the (im)mobilisation of potentially toxic elements.^7^ In the absence of oxygen, iron is the most abundant terminal electron acceptor for microbial respiration,^8,9^ and microbial Fe reduction may produce both aqueous ferrous iron (Fe(ii)) and oxidised organic carbon.^10^ Thus, in the absence of sulfide, which would favour the precipitation of iron sulfide minerals, it is likely that the ferrous carbonate mineral siderite is abundant in reducing soils and sediments.^11^ Indeed, the microbially catalysed formation of siderite has been widely observed in laboratory studies using a range of Fe sources and bacterial strains.^12–15^ Less is known concerning the formation and stability of siderite in natural environments, although it has been found in organic-rich marine sediments,^16^ clay-rich marine deposits,^17^ rice paddy soils,^18^ root iron plaques,^19^ peat soils,^20^ salt marshes,^21^ ferruginous lake sediments,^22^ and even highly acidic river water,^23^ suggesting that it is a naturally ubiquitous reservoir of mineral-bound Fe(ii).

Siderite is redox sensitive and its oxidation may facilitate the reduction of a range of contaminants including Se(iv),^24^ nitrite,^25^ Te(iv),^26^ Hg(ii),^27^ Tc(vii),^28^ Cr(vi),^29^ nitroaromatic compounds,^30^ and chlorinated aliphatics.^30^ Siderite is also of interest as a sorbent phase due to its high specific surface area, which ranges from 4.2 m^2^ g^−1^ (ref. 31) to 182 m^2^ g^−1^ (ref. 32) and has therefore been used to remove fluoride,^33^ lead,^34^ arsenic,^35^ uranium,^36^ and mercury^37^ from drinking water. Furthermore, the oxidation of siderite may produce reactive oxygen species, such as hydroxyl radicals, that are capable of oxidising highly recalcitrant contaminants^38,39^ and may influence CO_2_ release in thawing permafrost soils.^40^ However, despite the clear environmental importance, little is currently known about the oxidation of siderite under conditions representative of soils and sediments. Some studies suggest that synthetic siderite is extremely oxygen sensitive, and thus can be used as an oxygen indicator that changes from pure white to brown in seconds on contact with oxygen,^30^ whereas other work found that natural siderite was resistant to oxidation.^17^ The reason for this discrepancy is unclear, but could feasibly be due to the presence of organic matter or other potentially stabilising components associated with natural siderite.

Fe-chelating organic ligands, such as functional groups of natural organic matter and low molecular weight organic acids (LMWOAs) are ubiquitous in soils, sediments, and waters. The contribution of organic ligands to Fe redox processes and Fe mineral transformations are severalfold. For example, the presence of EDTA and hydroxamate/phenolic siderophores can accelerate the dissolution of iron oxyhydroxides,^41,42^ whereas organic matter of various classes has also been shown to stabilise ferrihydrite against Fe(ii)-catalysed transformation into more thermodynamically stable phases, most likely by hindering the growth of crystalline mineral phases.^43^ Citrate, nitrilotriacetic acid (NTA), and naturally extracted Pahokee Peat humic acid may promote the chemical oxidation of aqueous Fe(ii) by nitrite^44^ and similarly organically chelated Fe(ii) can accelerate or slow the rate of Fe(ii) oxidation^45–47^ depending on stability constants of the organic ligands.^48^ In the presence of an Fe-bearing mineral, organically complexed Fe can facilitate electron transfer to or from the solid phase and this redox buffering effect can, for example, promote the Fe catalysed production of reactive oxygen species.^49,50^ However, the influence of LMWOAs on siderite stability under oxic conditions is not well understood.

In this study, we used a series of batch experiments to determine the effect of organic ligands citrate, tiron, salicylate, and EDTA on siderite oxidation pathways, kinetics, and products. We selected these ligands to be analogues for LMWOAs that ubiquitously coexist with siderite in soils and sediments, and to contain a range of interesting functional groups representative of NOM, namely carboxylates (citrate, EDTA), aminopolycarboxylates (EDTA), catechols (salicylate, tiron), and sulfonates (tiron). To provide a full understanding of the impact of the organic ligands on the solid phase oxidation products, we also use chemical speciation modelling and a range of techniques for mineral characterisation including Mössbauer spectroscopy, Fe K-edge X-ray absorption spectroscopy (XAS), X-ray diffraction (XRD), and scanning transmission electron microscopy (STEM).

## 2 Materials and methods

### 2.1 Siderite synthesis

All chemicals used in this study are listed in the ESI (S1).† The synthesis and handling of all siderite suspensions was undertaken inside an anaerobic glovebox (MBRAUN, N_2_ atmosphere) with an O_2_ content <10 ppm. Anoxic solutions were prepared by purging with N_2_ for at least 2 hours and all glass/plasticware were introduced to the glovebox 24 hours before use to ensure removal of any remaining adsorbed oxygen. FeCl_2_ solution was prepared by dissolving metallic Fe(0) powder in 2 M HCl overnight under constant, gentle stirring at room temperature. The solution was filtered (0.22 μm, nylon), to ensure removal of any remaining Fe powder or ferric precipitates and the Fe concentration was checked using the colourimetric 1,10-phenanthroline assay, which has been previously described.^51^ Siderite was synthesised by slowly adding 50 mL of 1 M Na_2_CO_3_ solution to 50 mL of 1 M FeCl_2_ solution under magnetic stirring, which immediately produced a white precipitate consistent with siderite,^25^ and was then left to stir for 24 hours. The precipitate was then allowed to settle overnight, the supernatant decanted, replaced with ultra pure water (UPW) water (*ρ* ≥ 18.2 MΩ cm, Milli-Q) and then stirred for at least 4 hours. This process was repeated until the conductivity was measured as <200 μS cm^−1^ to ensure removal of excess sodium or chloride ions. We visually observed oxidation (a colour change from pure white to pale brown) in the washed precipitate if it was dried, even under anoxic conditions, therefore we stored the siderite as a suspension and determined the concentration in repeat digested samples (1 M HCl) using the 1,10-phenanthroline assay.^51^ Siderite purity was confirmed using XRD, Mössbauer spectroscopy, and XAS (detailed methods are provided in the next section) and despite previous work showing it is common for chukanovite to form alongside siderite,^52,53^ we saw no evidence of chukanovite or other mineral impurities in our synthesised material. In XRD, we observed moderately sharp peaks, corresponding to siderite with a crystallite size of ∼8 nm (Fig. S20a†). We used the same batch of siderite for all experiments and analyses and although the siderite was stored in suspension, further XRD measurements did not show any changes in crystallinity or other ageing effects.

### 2.2 Kinetic batch experiments

Anoxic stock solutions of the organic ligands, citrate, tiron, salicylate, and EDTA were prepared with UPW in 10 mM MOPS (3-(*N*-morpholino)propanesulfonic acid) buffer and adjusted to pH 7.5 ± 0.1 using 1 M NaOH or HCl. This pH was chosen to represent the pH of soils and sediments where siderite is abundant. The ligand concentration of the stock solutions was verified using high performance liquid chromatography (HPLC) as described in the next section. The chemical structures and p*K*_a_ values for the LMWOAs are provided in the ESI (S4).†

Batch reactors for kinetic analyses were prepared in triplicate, in 50 mL, wide bottomed serum vials with a total volume of 15 mL containing 14.8 mL of ligand stock solution and 200 μL of anoxic siderite stock suspension, to give final concentrations of 10 mM (LMWOA) and 2 g L^−1^ siderite (17 mM Fe(ii)). To investigate the influence of ligand concentration, further reactors were prepared containing 2 g L^−1^ siderite and 0.1 mM or 1 mM for citrate only. Control reactors were also prepared containing only 10 mM MOPS and siderite. A large headspace (∼35 mL) was provided to maximise the contact between the suspension and air during the oxidation experiments. The final pH was checked and if required, adjusted again to pH 7.5 ± 0.1. The initial Fe(ii) concentration in suspension in each reactor was determined using the 1,10-phenanthroline method.^54^ Reactors were foil wrapped to prevent photooxidation, crimp sealed, magnetically stirred at 150 min^−1^, and allowed to equilibrate for 24 h inside the anaerobic chamber. After 24 h, 1 mL filtered (0.45 μm, nylon) samples were taken, and the solids and filtrate saved, to determine the Fe(ii)/Fe(tot) ratio of the mineral solids and aqueous phase following the anoxic equilibration and to measure any siderite dissolution. Further identical, sacrificial reactors were prepared for Mössbauer, XAS, and XRD analyses and solids were combined from duplicate reactors for each measurement.

Reactors were then removed from the glovebox, stirred again at a rate of 150 min^−1^ using the same magnetic stir bar added inside the anaerobic chamber, and decrimped to commence oxidation. *p*CO_2_ and *p*O_2_ are assumed to be atmospheric.

To determine the Fe(ii)/Fe(tot) ratio of the bulk suspension, samples of 100 μL were periodically withdrawn using a pipette with a cut-off tip and immediately digested in 1 mL of 5 M HCl. The 1,10-phenanthroline assay^54^ was used to determine the Fe(ii)/Fe(tot) ratio. Additionally, the Fe concentration and Fe(ii)/Fe(tot) ratio of the aqueous phase was determined by taking samples of 1 mL at regular intervals, filtering (0.45 μm, nylon) and immediately acidifying before analysis with the 1,10-phenanthroline assay.^54^ The Fe(ii)/Fe(tot) ratio of the solid phase was then calculated by calculating the difference between the bulk and aqueous phases. A further portion of the filtrate was used for HPLC quantification of the organic ligand concentration. The solids were also kept and immediately transferred into the anaerobic chamber for drying and storage until mineral characterisation. All samples were stored in the dark, in the fridge (4 °C) prior to analysis. Samples for Fe speciation were analysed within 48 h and for LMWOA quantification within 5 days.

### 2.3 Analytical methods

Further details on some of the analytical methods (HPLC, Mössbauer spectroscopy, X-ray diffraction, X-ray absorption spectroscopy) described in this section are found in the ESI Section S3.† The 1,10-phenanthroline assay was used to quantify Fe(ii)/Fe(tot) ratios as previously described.^51,54^ To ensure that the LMWOAs did not influence the accuracy of the method, we also tested Fe calibration standards containing the respective ligands and did not observe any significant difference in the results. The concentration of LMWOAs citrate, tiron, salicylate, and EDTA was quantified using an Agilent 1100 series HPLC instrument, equipped with a DAD-detector, with an Agilent ZORBAX Eclipse XDB-C18 column (4.6 mm × 150 mm, 5 μm).^55^ Fe Mössbauer spectroscopy (WissEl, Wissenschaftliche Elektronik GmbH equipped with a closed-cycle He cryostat (Janis Research SHI-850-5)) was used to determine the reduction extent and the nature of the siderite and associated oxidation products. Mineral identification was performed with powder X-ray diffraction (XRD, D8 Advance, Bruker) with Rietveld analysis (Topas, Bruker). Bulk Fe K-edge (7112 eV) X-ray absorption spectroscopy (XAS) with linear combination fitting (LCF) of X-ray absorption near edge structure (XANES) and extended X-ray absorption fine structure (EXAFS) spectra was used to further characterise our synthetic siderite and oxidation products. Finally, the synthetic siderite and oxidation products were characterised on a scanning transmission electron microscope (STEM, 2700Cs, Hitachi) operated at 200 kV. Samples were prepared in the anaerobic chamber by suspending approximately 2 mg of sample material in UPW water, which was drop-deposited onto a 200-mesh Cu grid with a pervious carbon support film (SPI supplies, USA).

### 2.4 Kinetic and speciation modelling

To better understand siderite oxidation, we modelled the kinetics of the reaction using a pseudo-first order kinetic rate law (eqn (1)), which was solved for our triplicate data using the Ode15s differential equation solver method in Matlab.^56^ The model was based on the digested suspension samples and therefore represents the bulk (*i.e.* solids and aqueous) oxidation kinetics.1
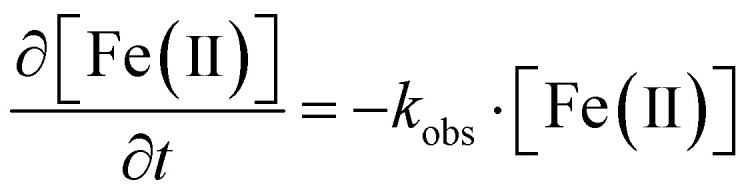


The equilibrium speciation of the LMWOAs and Fe-LMWOA complexes was calculated for all reactors using PHREEQC based on the measured concentrations of Fe(ii), Fe(iii), and LMWOAs. Stability constants were taken from the IUPAC stability constants database^57^ as listed in Table S3† (p*K*_a_ values for the ligand species are also provided in Table S2† and corrected for the specific ionic strength in our reactors (∼0.1)).

The stability constants were also used to calculate the standard one-electron reduction potentials of the Fe-LMWOA complexes, using the following version of the Nernst equation, as previously described.^55^2
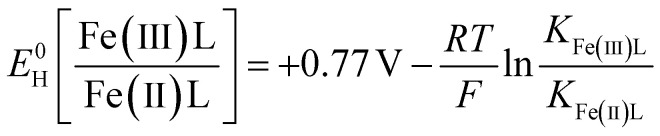


*E* ^0^_H_ is the standard reduction potential of the Fe species *vs.* the NHE, *K*_Fe(*x*)L_ is the stability constant for the Fe species. *R*, *T* and *F* are the molar gas constant (8.3145 J mol^−1^ K^−1^), temperature (298 K) and Faraday constant (96.485 kJ mol^−1^).

## 3 Results

### 3.1 Aqueous speciation and ligand-controlled dissolution

For siderite alone in buffer and siderite in the presence of salicylic acid, we did not measure any aqueous Fe during the course of our experiments (lowest Fe calibration standard = 1 μM, Table S5†) either during the anoxic equilibration time (24 h) or during the oxidation phase, indicating that no dissolution of siderite occurred. In the presence of EDTA, 7.9 ± 0.4 mM of Fe (100% Fe(ii), Fig. S3c and Table S5†) was released to the aqueous phase during the anoxic equilibration, decreasing to 3.4 ± 0.4 mM Fe (46% Fe(ii)) after 24 hours of oxidation, indicating that some of the dissolved Fe(ii) had either been sorbed to the solid phase or had re-precipitated after the onset of oxidation. With 10 mM citrate, 3.2 ± 0.6 mM Fe was released to solution during the anoxic phase, increasing to 9.8 ± 0.3 mM Fe (14% Fe(ii)) after 24 h oxidation (Fig. S3a and Table S5†). In the case of tiron, we found that 2.3 ± 0.1 mM Fe (100% Fe(ii)) was initially released to the aqueous phase, which increased to 5.3 ± 0.4 mM Fe (77% Fe(ii)) following oxidation (Fig. S3b and Table S5†).

Aqueous concentrations of the LMWOAs over time were measured using HPLC and the carboxylate containing ligands citrate and EDTA sorbed to the siderite before the onset of oxidation (1.2 mM and 0.9 mM respectively, Fig. S5†) with the sorbed concentration remaining approximately stable over the course of the oxidation experiment for citrate, and increasing to 2.9 mM for EDTA. In the reactors containing tiron and salicylate, the ligands did not sorb during the anoxic equilibration period. However, after the onset of oxidation the sorbed concentration increased reaching a maximum of 1.6 mM and 1.7 mM, respectively, after 24 hours (Fig. S5†).

Aqueous speciation modelling showed that most of the aqueous Fe was organically complexed (Fig. S2†) and formed soluble Fe–ligand complexes and therefore remained in the aqueous phase upon oxidation with the exception of reactors containing 10 mM citrate, which contained up to 0.24 mM Fe(OH)_2_^+^, 0.08 mM Fe(OH)_3_, and 0.54 mM Fe^2+^ (9% of aqueous Fe).

### 3.2 Siderite oxidation kinetics

To investigate siderite oxidation kinetics in the presence of LMWOAs, we measured the Fe(ii)/Fe(tot) ratios over time in the bulk suspension, aqueous phase, and solid phase. For simplicity, in the following section we present the bulk data unless stated otherwise. Data for aqueous Fe(ii) and solid Fe(ii) are shown in Fig. S4 and S6.†

**Fig. 1:**
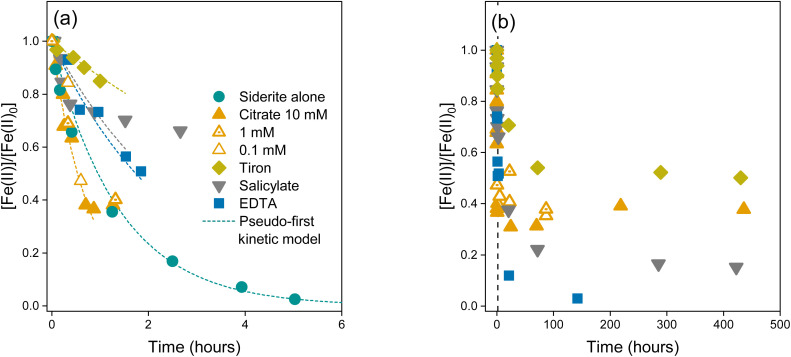
Oxidation kinetics of bulk Fe(ii), stirred in oxygen saturated suspensions at pH 7.5 (a) over the course of 6 hours with dashed lines showing a pseudo-first order kinetic fit and (b) over 500 hours. Markers represent data from one replicate experiment and the dashed lines show a pseudo-first order kinetic fit. We include the data for salicylate (grey) to show that the pseudo-first order kinetic model does not adequately fit the observed kinetics. The black dashed line in panel (b) is placed at 6 hours to show the extent of panel (a).

In the absence of a LMWOA, the synthetic siderite suspension at pH 7.5 was completely oxidised in under 6 hours (Fig. 1a), as has been previously observed for synthetic siderite.^58^ The oxidation kinetics were best described using a pseudo-first order rate law (log *k*_obs_ = −0.14 h^−1^), suggesting that the siderite underwent bulk, homogeneous oxidation. In contrast, in the presence of all the LMWOAs we investigated, the oxidation of siderite-Fe(ii) occurred at a slower rate. Our text here describes the bulk oxidation rate of the suspension including both mineral-bound and aqueous Fe. For citrate (0.1–10 mM), salicylate, and tiron we observed an initial rapid drop in [Fe(ii)]/[Fe(ii)_0_], after which the Fe(ii) content plateaued and continued to drop at a much slower rate. We did not observe complete Fe(ii) oxidation before our last measurement point at 90 hours for 0.1 mM and 1 mM citrate and 450 hours for 10 mM reactors (Fig. 1b), and we did not observe a change in oxidation kinetics in the presence of different concentrations of citrate. For EDTA, we did not observe a plateau phase and we observed complete oxidation of the Fe(ii) in approximately 150 hours. For citrate, tiron, and EDTA, the oxidation kinetics followed a pseudo-first order kinetic rate law for approximately the first 1–2 hours prior to the plateau phase of the reaction (Fig. 1a, dashed line, Table S6,† log *k*_obs_ = −0.08–0.12 h^−1^, −0.40 h^−1^, and −0.84 h^−1^ respectively). Despite similarly observing initial rapid oxidation kinetics in the presence of salicylate, we could not model the kinetics during this period according to any kinetic rate law.

To ensure the accuracy of our kinetic data, based on dissolution of the bulk suspension in 5 M HCl, we also determined the Fe(ii)/Fe(tot) ratio of the solid phase alone using Mössbauer spectroscopy, LCF of Fe K-edge XANES spectra, and calculated the Fe(ii)/Fe(tot) ratio of the solid phase alone in the digested samples by correcting for the measured dissolution and aqueous Fe(ii)/Fe(tot) ratios (Table S7/Fig. S6†). Samples for Mössbauer analysis were taken after 1 hour and 24 hours oxidation, XAS after 24 hours, and for most sampling points we found good agreement between the digestion and solid phase measurements. We observed a discrepancy for citrate at the 1 hour sampling point. We attribute this to the samples being taken during the rapid initial drop phase, where slight errors in the timing of the sample collection will lead to larger error. However, the bulk digestion, after correction for what was measured in the aqueous phase, underestimated the solid Fe(ii)/Fe(tot) ratio by 21% compared to Mössbauer fits and 27% compared to XANES for citrate sampled after 24 hours. The bulk digestion also overestimated the Fe(ii)/Fe(tot) ratio by 34% compared to Mössbauer fits and 42% compared to XANES for tiron sampled after 24 hours (Table S7/Fig. S6†). The reason for this discrepancy is unclear. One possibility is that this is a result of an artefact of the 1,10-phenanthroline method despite using standards containing the LMWOAs. Alternatively, it could be experimental error as although our measurements were carried out in triplicate with the same batch of siderite and ligand stocks, separate, sacrificial reactors were used for kinetic analyses, XAS, and Mössbauer analyses.

### 3.3 Mössbauer spectra of siderite

To aid our understanding of how the presence of LMWOAs influence siderite oxidation, we also characterised the initial siderite and solid Fe products formed from siderite oxidation in the presence and absence of organic ligands using Mössbauer spectroscopy, LCF of Fe K-edge EXAFS spectra, and XRD.

As only a few studies have previously used Mössbauer spectroscopy to characterise pure siderite, especially at low measurement temperatures we present spectra for all measurement temperatures here. The Mössbauer spectra of our pure synthetic siderite measured at 140 K and 77 K appears as a doublet with a uniquely narrow Quadrupole Splitting (QS, 2.04 mm s^−1^, Fig. S7a, b and Table S8†), consistent with existing literature values.^59,60^ However, we could only achieve a satisfactory fit for our spectra by incorporating a second ferrous doublet with a QS of 2.76 mm s^−1^. Despite previous work showing that chukanovite is a common byproduct of siderite synthesis, we observed no evidence of chukanovite or other mineral impurities in XRD. Thus, the second doublet is likely to be either intrinsic to our siderite or may suggest that some Fe(ii) remained sorbed to the siderite after synthesis, despite our extensive washing procedures.

At 4 K, the spectra comprises what appears to be two overlapping octet phases (Fig. S7c†). We were unable to satisfactorily fit this spectra, however we present the data as to our knowledge no published spectra currently exist, and to act as further confirmation that the ferrous phase observed at higher temperatures comprises siderite by identifying characteristic peaks at *v* = −1.33 mm s^−1^, −0.54 mm s^−1^, −0.08 mm s^−1^, 0.81 mm s^−1^, 1.99 mm s^−1^, 3.41 mm s^−1^, 5.29 mm s^−1^, which are not representative of other common ferrous minerals including ferrous hydroxide, green rust, chukanovite, or magnetite.^61^

### 3.4 Oxidation products

**Fig. 2:**
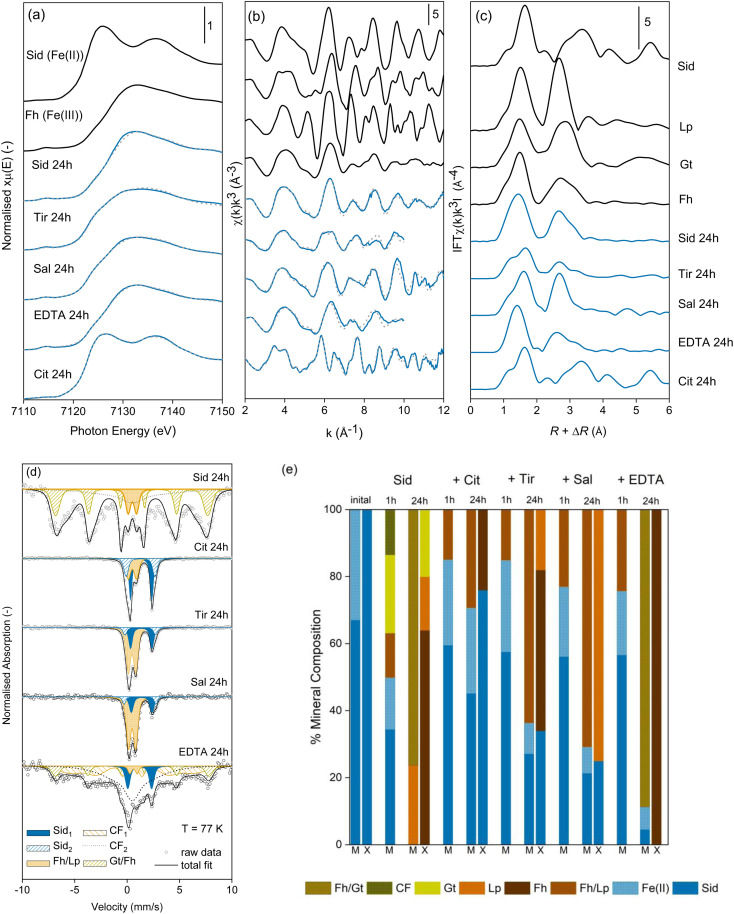
Spectra of the solid phase after 24 hours oxidation: (a) linear combination fits (LCF) of the Fe K-edge XANES spectra, (b) LCF of the Fe K-edge EXAFS spectra, (c) Fourier transforms of the Fe K-edge EXAFS spectra, (d) Mössbauer spectra and fits measured at 77 K, and (e) percentage mineral composition after 1 hour and 24 hours oxidation as measured by Mössbauer (M) and 24 hours as calculated from LCF fits of the EXAFS spectra (X). Fits of the XANES and EXAFS spectra are represented by dashed lines. Abbreviations: Sid = siderite, Gt = goethite, Lp = lepidocrocite, Fh = ferrihydrite, CF = collapsed feature/poorly ordered phase, Cit = citrate, Tir = tiron, and Sal = salicylate.

In the absence of LMWOAs, upon exposure to air, siderite was rapidly oxidised to Fe(iii) (oxyhydr)oxide phases (Fig. 2). After 1 hour stirring in air, Mössbauer spectra measured at 77 K (Fig. 2d) showed that 50.0% of the Fe remained as two Fe(ii) doublets, likely to comprise siderite due to the characteristically narrow QS of the inner doublet. The remainder of the spectral area comprised a ferric doublet (12.6%) likely to consist of ferrihydrite and/or lepidocrocite, a sextet (21.5%) likely to comprise goethite, and a collapsed feature (15.7%), which could comprise either poorly crystalline goethite^62^ or ferrihydrite with features consistent with 6-line ferrihydrite.^63^

After 24 hours, Fe(ii) was no longer visible in the Mössbauer spectra (Fig. 2d) and at 77 K approximately 8% of the area remained as an Fe(iii) doublet, with the remaining area comprising a collapsed, poorly ordered sextet. At a measurement temperature of 4 K the collapsed features appear fully ordered and we fit three sextets to the spectrum (Fig. S9c†). One sextet corresponds to lepidocrocite (19% of the spectral area) and two sextets (81% of the spectral area) have the parameters in the range of both poorly crystalline goethite and 6-line ferrihydrite^64^ and we refrain from distinguishing between them based on this data.^63,65^ Indeed, our XRD measurement of the sample after 24 hours shows broad goethite peaks which would correspond to nano-goethite (Fig. S19b†) and Rietveld fitting estimates a crystallite size of ∼4 nm. LCFs of the Fe K-edge EXAFS spectra of the oxidation products in the absence of LMWOAs after 24 hours also indicated the formation of goethite (20%), ferrihydrite (64%), and lepidocrocite (16%, Fig. 2). The Mössbauer and LCF fits of the EXAFS spectra at 24 h showed generally good agreement (Fig. 2e).

In the presence of all LMWOAs tested, the formation of Fe(iii) (oxyhydr)oxide oxidation products occurred at a slower rate consistent with the retarded oxidation kinetics. Citrate inhibited the oxidation rate of siderite the most followed by tiron, then salicylate, then EDTA. For all LMWOAs studied, siderite remained the dominant fraction after 1 hour (75–85%) and was preserved even after 24 hours exposure to air (11–71%) (Fig. S10–S18†). For citrate, tiron, and salicylate no goethite formation was observed in either Mössbauer or LCF fits of the EXAFS spectra and the products comprised ferrihydrite or lepidocrocite (Fig. 2e). Mössbauer measured at 77 K did not allow differentiation between ferrihydrite and lepidocrocite but the LCF fits of the Fe K-edge EXAFS spectra and XRD analyses suggest the product was predominantly ferrihydrite in the presence of citrate and tiron, and lepidocrocite in the presence of salicylate. The XRD of siderite in the presence of salicylate shows rather broad lepidocrocite peaks suggesting that the lepidocrocite is poorly crystalline and Rietveld fitting suggests a crystallite size of ∼9 nm (Fig. S19e†). For 0.1 mM citrate peaks are observed consistent with 6-line rather than 2-line ferrihydrite (Fig. S19g†). For siderite in the presence of EDTA after 24 hours, the Mössbauer spectra measured at 140 K and 77 K again contained poorly ordered features comprising approximately 90% of the spectral area. Due to the low proportion of siderite remaining in the sample, we were able to fit the 4 K spectra, where 92% of the spectral area comprises two sextets with parameters in the range for both ferrihydrite and goethite. Again, we do not distinguish between them based on this data, however, again the presence of collapsed features at 140 K and 77 K could imply the presence of nano-goethite.^62^ LCF fits of the EXAFS spectra only suggested the presence of ferrihydrite and our XRD data (Fig. S19f†) closely resembles 6-line ferrihydrite with a weak peak (17.6° 2*θ*) that suggests a very small amount of nanocrystalline lepidocrocite is present.

### 3.5 Electron microscopy

**Fig. 3:**
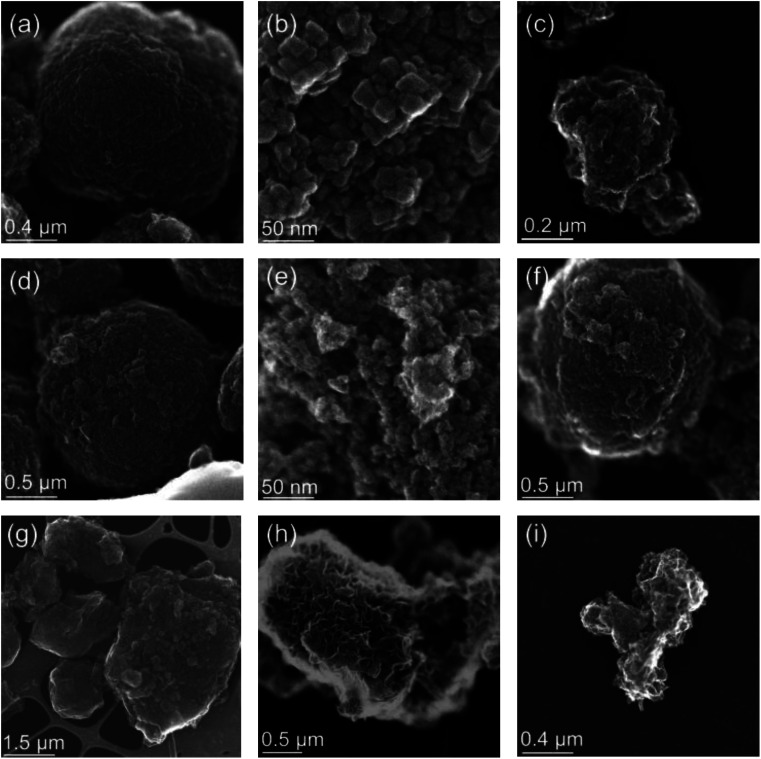
STEM images of (a) our synthetic siderite comprising spherical particles, (b) the surface of the siderite spheres showing polyhedral primary particles with a diameter of approximately 10 nm, (c) siderite oxidation product comprising ferrihydrite, lepidocrocite, and goethite (d) siderite that has been oxidised in the presence of citrate retaining a spherical structure, (e) the surface of the spherical particles formed in the presence of citrate, (f) siderite oxidised in the presence of EDTA, (g) tiron, (h) salicylate showing larger particles, and (i) salicylate showing smaller particles.

In order to better understand how the presence of LMWOAs affects the oxidation of siderite we also characterised the morphology of siderite and its oxidation products, with and without the presence of LMWOAs, using transmission electron microscopy. The synthetic siderite formed large spherical particles with a diameter of approximately 1 μm (Fig. 3a) comprising polyhedral subunits with a size of approximately 10–20 nm (Fig. 3b), consistent with previous studies.^66–70^ Following oxidation in the absence of LMWOAs, the material transformed into small (∼200 nm) particles consistent with ferrihydrite aggregates^71^ (Fig. 3c). We did not observe any lath-like particles or large, well-defined needle-like particles indicating that the lepidocrocite and goethite observed in the XRD, EXAFS, and Mössbauer analyses were nanocrystalline.

Interestingly, following 24 hours of oxidation, in the presence of citrate (Fig. 3d), EDTA (Fig. 3f), and to a lesser extent tiron (Fig. 3g), we still observed spherical particles similar to the original siderite with a diameter of approximately 1 μm even though our Mössbauer analyses suggests that the products comprise 30%, 89%, and 64% ferrihydrite respectively. For EDTA and tiron some smaller particles were also visible. However, when the spherical particles are viewed at greater magnification, the polyhedral subunits forming the original siderite spheres are no longer visible but rather the surface of the spheres appear to comprise aggregates of nanosized particles, which are more consistent with ferrihydrite (Fig. 3e). However, in the presence of salicylate angular particles of mixed size (<0.2–1 μm, Fig. 3h and i) are visible, although our Mössbauer suggests that this still comprises 29% siderite.

## 4 Discussion

### 4.1 Stabilisation of siderite in the presence of LMWOAs

Our Mössbauer spectra and LCF fits of the Fe K-edge EXAFS spectra showed that all the LMWOAs studied preserved siderite against oxidation in the order citrate > tiron > salicylate > EDTA. Most of the oxidation occurred in the first 1–2 hours after which the reaction rate decreased. The presence of organic ligands at the mineral surface likely influenced the mechanism of oxidation and/or the composition of the Fe oxidation products formed. The adsorption of organic matter to iron minerals has previously been attributed to surface complexation–ligand exchange reactions and can occur for both carboxyl and catechol functional groups.^72,73^ At the surfaces of carbonate minerals, carboxylic acids form mono- or bidentate complexes with adjacent cation sites^74^ and catechol and sulfonate groups only interact weakly with the carbonate mineral surface^75^ therefore explaining the preferential sorption of carboxylate-containing citrate and EDTA over the catechol-containing salicylate and tiron under anoxic conditions. Although salicylate contains a carboxyl group, it is likely that steric arrangement prevented this mechanism. However, the point of zero charge (PZC) previously reported for siderite in literature varies from 4.0 to 7.2;^76–78^ thus, it is likely to be negatively charged under our experimental conditions (pH 7.5), whereas iron (oxyhydr)oxide phases tend to have higher PZC values (7.2–7.8).^79^ Therefore, the oxidation products are more likely to be positively charged in our reactors and have a higher capacity to sorb the negatively charged LMWOAs, explaining the sorption of salicylate and tiron only under oxic conditions. However, we did not observe a relationship between the rate and extent of LMWOA sorption and siderite oxidation kinetics, indicating that sorption extent alone does not explain the observed stabilisation of siderite.

Our finding that the structure of spherical particles is retained after oxidation in the presence of LMWOAs is intriguing. In the absence of an organic ligand, siderite oxidation is thought to proceed through the oxidation of surface Fe(ii)–hydroxyl groups at circumneutral pH, which precipitate directly as ferrihydrite.^80^ The ferrihydrite may then undergo Fe(ii)-catalysed transformation to the more thermodynamically stable Fe(iii)-minerals lepidocrocite and goethite.^81^ On the basis of our STEM images, in the absence of organic ligands the large siderite spheres break down upon oxidation to form smaller Fe(iii) (oxyhydr)oxide particles (Fig. 3). However, in the presence of LMWOAs, the spherical structure is retained. This effect is most pronounced for citrate and EDTA, less so for tiron and even less for salicylate. We hypothesise that the sorption of organic ligands encourages the retention of the Fe oxidation products at the mineral surface, allowing the formation of an Fe(iii) (oxyhydr)oxide layer around the siderite core. Indeed, a previous study found that the presence of ascorbic acid may enhance the aggregation of siderite particles under anoxic conditions, encouraging the retention of spherical particles.^66^ In the cases of salicylate, tiron, and EDTA we observed either only amorphous product particles (salicylate) or a mixture of spheres and small particles (tiron, and to a lesser extent EDTA). For salicylate and tiron, we did not observe any sorption of the LMWOAs to the siderite before the onset of oxidation. Thus, we suggest that limited particle breakdown, as observed for siderite in the absence of organic ligands, occurs before sorption of the LMWOAs is established. Alternatively, as ferrihydrite is known to form stable aggregates,^82^ it is possible that the formation of lepidocrocite as an oxidation product in the presence of tiron and salicylate contributed to the further particle breakdown observed.

The formation of an Fe (oxyhydr)oxide layer at the siderite surface alone is not sufficient to explain the inhibited siderite oxidation, as ferric Fe can also rapidly oxidise siderite.^80^ However, we hypothesise that oxidation is hindered by the presence Fe(iii)–organic ligand complexes at the surface of the siderite core, which inhibit electron transfer as all the LMWOAs studied here are considerably more stable as Fe(iii)–ligand species than Fe(ii)–ligand species (Table S3†) and exhibit lower redox potentials (*E*_H_) than Fe (oxyhydr)oxides (Table S4,† the contribution of *E*_H_ of the individual LMWOAs is discussed in the next section). Thus, we hypothesise that siderite oxidation is inhibited by the formation of a passivation layer comprising both Fe(iii)–organic ligands and Fe (oxyhydr)oxides. Our data indicates that the majority of the outer layer comprises Fe (oxyhydr)oxides as we did not observe organically complexed Fe in our EXAFS spectra (detection limit 5%). It is possible that during the initial rapid phase of oxidation the mechanism of oxidation proceeds similarly to siderite in the absence of LMWOAs, explaining the similar pseudo-first order kinetics. During this phase we also observe rapid dissolution (citrate, tiron) or resorption (EDTA) of Fe from/to siderite indicating that dynamic LMWOA sorption and Fe dissolution/sorption occurs at the mineral surface (Fig. S3†). After the first 1–2 hours of oxidation, the rate of both Fe dissolution/sorption and oxidation stabilises (Fig. 1 and S3†) suggesting that a passivation layer would be well established and stable enough to hinder oxidation of the siderite core. For salicylate, where we did not observe dissolution or pseudo-first order kinetics in the initial phase we suggest this deviation is due to concurrent particle breakdown and passivation layer formation.

### 4.2 Assessing the influence of the type of LMWOA

The nature of the LMWOA also influenced the extent to which siderite was stabilised against oxidation, with only 7/11% Fe(ii) (XANES/Mössbauer) remaining in the presence of EDTA after 24 hours oxidation, compared to the 77/71% (XANES/Mössbauer) that remained in the presence of citrate. The organic ligands we investigated complex Fe through either carboxylate (citrate, EDTA), or catecholate (tiron, salicylate) functional groups. As the carboxylate ligands likely form mono- or bidentate complexes^74,75^ at the siderite surface, this leaves both free negatively charged carboxyl groups for both EDTA and citrate at the surface, favouring the retention of Fe(iii) and stabilising the passivation layer. Tiron may also form mono- or bidentate complexes and the sulfonate groups create excess negative charge. As salicylate contains only one hydroxyl group it likely forms an uncharged monodentate complex with siderite, further explaining the increased particle breakdown observed in the presence of salicylate. However, the presence of the aromatic ring may alter charge density at the siderite surface^83^ still allowing the formation of a passivation layer rather than complete particle breakdown and oxidation observed in the absence of a LMWOA. However, although the type of functional group explains the extent of retention of the original siderite spherical structure, we do not see a relationship with the extent and rate of oxidation.

Therefore, we also considered the stability constants of each ligands species with regards to both Fe(ii) and Fe(iii) (Table S3†) and therefore the *E*_H_ values^55^ for the most important redox couples (Table S4†). As we hypothesise that electron transfer is hindered by the presence of thermodynamically stable Fe(iii)–organic ligand complexes at the surface of the siderite core, it stands to reason that the most stable complexes (*i.e.* with the lowest *E*_H_) should inhibit oxidation the most. However, we do not see a relationship with the *E*_H_ values of the aqueous Fe-complexes although it is unclear whether these represent the coordination of the complexes at the siderite surface.

As we cannot relate the extent of siderite stabilisation to either the type of functional group or the stability of the Fe–organic ligand complexes, we hypothesise that the extent to which siderite surface sites are occupied by Fe(iii)–organic ligands may explain the behaviour we observed. Although we cannot calculate this on the basis of our data, as we observe a plateau in oxidation kinetics for citrate, salicylate, and tiron but not for EDTA we theorise that non-organic Fe (oxyhydr)oxide surface sites exist at the surface of the siderite core. This would explain why in the presence of EDTA slow but complete oxidation of siderite occurs, whereas not in the presence of the other LMWOAs during the time course of our experiments. Indeed, previous work has indicated that as EDTA is a bulky molecule with excess negative charge, it may be unable to fill adjacent cation sites at a carbonate surface.^75^ However, further work investigating both the surface coverage and surface speciation of LMWOAs would be required to confirm this hypothesis.

### 4.3 Inhibition of crystalline Fe oxidation products

Our results show that in the absence of LMWOAs, siderite rapidly oxidised to form relatively amorphous Fe oxidation products including nanocrystalline lepidocrocite and goethite. This supports previous work that has shown that siderite oxidation products comprise phases with a very high sorption capacity for contaminants such as arsenic.^35^ Our data show that the presence of LMWOAs suppressed the formation of goethite in favour of ferrihydrite or lepidocrocite, which are also known to have a high surface area and comprise important sorbents for nutrients and contaminants in soil and groundwater environments.^84,85^ It is widely accepted that the presence of organic matter and organic ligands inhibit the Fe(ii)-catalysed transformation of ferrihydrite to more crystalline iron minerals such as lepidocrocite and goethite.^86–88^ A proposed mechanism for this inhibition is the complexation of either aqueous Fe(ii) by the organic ligands^88,89^ or of labile Fe(iii) intermediates.^90^ Indeed, our speciation calculations show that most of the aqueous Fe(ii) is complexed by the ligand phases (Fig. S2†). Another possibility is that the surface adsorbed organic ligands may inhibit the growth of goethite by oriented attachment.^86,91^ Furthermore, as the resulting ferrihydrite is retained at the surface of the siderite sphere, there are less ferrihydrite surfaces exposed to the solution to allow further transformation to occur. However, our data cannot rule out direct precipitation of lepidocrocite and goethite. Indeed, lepidocrocite and goethite have been observed to form upon the oxidation of aqueous Fe(ii)^92,93^ and goethite has been observed as the sole oxidation product of siderite.^35^ For salicylate, where the only oxidation product formed was lepidocrocite, direct precipitation is credible and would explain the poorly crystalline nature of the formed lepidocrocite. However, for tiron, both ferrihydrite and lepidocrocite are formed. As breakdown of the large siderite spheres into smaller particles occurred in the presence of both salicylate and tiron, this could support that ferrihydrite initially forms and transforms into lepidocrocite due to the increased surface area resulting from the particle breakdown in the presence of salicylate and tiron in contrast to citrate and EDTA. This hypothesis is further supported by the formation of more crystalline 6-line ferrihydrite in the presence of the lower 0.1 mM concentration of citrate, where there will be a lower concentration of ligand at the particle surface, allowing for ferrihydrite to transform to more stable, crystalline phases. However, direct goethite precipitation would likely be inhibited by the presence of LMWOAs through the inhibition of crystal growth through oriented attachment.^91^

## 5 Conclusions and environmental relevance

Our results show that in the presence of LMWOAs, siderite is stabilised against oxidation. The process is complex and likely involves several concurrent reactions including siderite/oxidation product dissolution, oxidation of aqueous Fe(ii)–LMWOA complexes, the formation of an Fe (oxyhydr)oxide surface layer, and the Fe(ii)-catalysed transformation of Fe (oxyhydr)oxide oxidation products and it is not possible to discern the contributions of each process towards the overall oxidation kinetics on the basis of our data. However, we suggest on the basis of our STEM images that siderite is stabilised due to the formation of an Fe (oxyhydr)oxide/Fe(iii)–organic ligand passivation layer. In soils and sediments, siderite may therefore be more stable in the presence of oxygen than has previously been accounted for and may persist in natural environments such as periodically oxic soils and sediments, including tidal areas and rice paddies as it is likely more stable in these natural environments, where DOC is ubiquitously present.^94^

This has implications for many of the proposed applications of siderite. For example, oxidised siderite has been proposed as an efficient sorbent for arsenic due to the formation of products with extremely high surface areas.^35^ However, the rate of siderite oxidation, and the composition/surface area of the oxidation products formed may be quite different in the presence of naturally occurring organic ligands than has been determined in laboratory model studies. Nonetheless, as we show that in the presence of LMWOAs the formation of ferrihydrite and nanocrystalline lepidocrocite is favoured, which may have even larger surface areas than the nanocrystalline goethite formed in the absence of LMWOAs, our data supports the use of siderite oxidation products as highly effective sorbent phases. However, studies that propose the use of siderite oxidation as a source of electrons for the reduction of contaminants,^24,25,27–30,95–97^ or for the production of hydroxyl radicals from siderite oxidation^38,39^ may not be relevant in complex biogeochemical environments such as soils, sediments, and groundwaters, as siderite might not be as an effective a source of electron equivalents as anticipated in periodically oxic environments such as soils and sediments where organic matter is ubiquitously present.

## Data availability

All data required to understand the manuscript are included in the ESI.† Additionally, full raw data used to compile this manuscript has been published on the Zenodo repository and is available with DOI: https://doi.org/10.5281/zenodo.12189023.

## Author contributions

K. R. – conceptualisation, methodology, investigation, data analysis, visualisation, writing – original draft; L. T. A. – investigation (XAS), writing – review & editing; R. Kaegi – investigation (electron microscopy), writing – review & editing; R. Kretzschmar – conceptualisation, funding acquisition, writing – review & editing.

## Conflicts of interest

There are no conflicts to declare.

## Supplementary Material

EM-027-D4EM00363B-s001
