# Supplementary material for: Low molecular weight organic acids stabilise siderite against oxidation and influence the composition of iron (oxyhydr)oxide oxidation products
Source: Environ Sci Process Impacts. 2024 Nov 25;27(1):133–45. doi: 10.1039/d4em00363b (PMC11606451; doi:10.1039/d4em00363b)
Supplement: EM-027-D4EM00363B-s001 [file EM-027-D4EM00363B-s001.pdf]

Supplementary Information (SI) for Environmental Science: Processes & Impacts.
This journal is © The Royal Society of Chemistry 2024

Supporting Information to: Low molecular weight organic acids stabilise siderite against oxidation and influence the composition of iron (oxyhydr)oxide oxidation products

Katherine A. Rothwell*[1†], Laurel K. ThomasArrigo[1‡], Ralf Kaegi[2] and Ruben Kretzschmar[1]

[1]Soil Chemistry Group, Institute of Biogeochemistry and Pollutant Dynamics, Department of Environmental Systems Science, ETH Zürich, CHN, Universitätstrasse 16, CH-8092, Zürich, Switzerland

[2]Eawag, Swiss Federal Institute of Aquatic Science and Technology, Überlandstrasse 133, CH-8600 Dübendorf, Switzerland

[†]Present address: School of Earth Sciences, Wills Memorial Building, Bristol, BS8 1RJ, United Kingdom

[‡]Present address: Environmental Chemistry Group, Institute of Chemistry, University of Neuchâtel, Avenue de Bellevaux 51, Neuchâtel, Switzerland

Pages: 37
Figures: 19
Tables: 12

*k.rothwell@bristol.ac.uk

Contents

S1 Chemicals and Reagents **S3**

S2 Siderite Synthesis **S3**

S3 Detailed Analytical Methods **S3**
S3.1 High Performance Liquid Chromatography (HPLC) S3
S3.2 Mössbauer Spectroscopy S4
S3.3 X-Ray Diffraction S4
S3.4 X-Ray Absorption Spectroscopy S5

S4 Organic Ligand Properties **S6**

S5 Aqueous Speciation of Fe **S10**

S6 Fe Dissolution **S11**
S6.1 Aqueous Fe(II) S12

S7 Ligand Sorption **S14**

S8 Oxidation Kinetics **S15**

S9 Mössbauer Spectra and Fits **S18**

S10 X-Ray Absorption Spectroscopy LCF-fitting **S30**
S10.1 XANES S30
S10.2 EXAFS S31

S11 X-Ray Diffraction Results **S34**

S1 Chemicals and Reagents

All chemicals and reagents used in this study were of analytical grade or higher and purchased from Merck (Fe metal, hydrochloric acid, 1,10-phenanthroline, sulfuric acid, ammonium acetate, EDTA, citrate, tiron, methanol (LiChromasolv grade), orthophosphoric acid, potassium dihyrogen phosphate, boron nitride, ferrous ammonium sulfate), or Fluka (sodium carbonate, salicylate), or Sigma Aldrich (glacial acetic acid).

S2 Siderite Synthesis

The synthesis and handling of all siderite suspensions was undertaken inside an anaerobic glovebox (MBRAUN, N_2 atmosphere ≥99.995%) with an O_2 content < 10 ppm. Anoxic solutions were prepared by purging with N_2 for at least 2 hours and all glass/plasticware were introduced to the glovebox 24 hours before use, to ensure removal of any remaining sorbed oxygen. $FeCl_2$ solution was prepared by dissolving metallic Fe(0) powder in 2 M HCl overnight under constant, gentle stirring at room temperature. The solution was filtered (0.22 μm, nylon), to ensure removal of any remaining Fe powder or ferric precipitates and the Fe concentration was checked using the colourimetric 1,10-phenanthroline assay, which has been previously described.[1, 2] Siderite was synthesised by slowly mixing 50 mL of 1 M $FeCl_2$ solution with 50 mL of 1 M Na_2CO_3 solution, which immediately produced a white precipitate [3], which was left to stir for 24 hours. The precipitate was then allowed to settle overnight, the supernatant decanted, replaced with ultra pure water (UPW) water ($\rho > 18$ MΩ cm, Milli-Q) and then stirred for at least 4 hours. This process was repeated until the conductivity was measured as < 200 µS cm^{-1} to ensure removal of excess sodium or chloride ions. We visually observed oxidation (a colour change from pure white to pale brown) in the washed precipitate if it was dried, even under anoxic conditions, therefore we stored the siderite as a suspension and determined the concentration in repeat digested samples (1 M HCl) using the 1,10-phenanthroline assay [1]. The siderite was characterised using XRD (detailed methods are provided in the next section) and we observed moderately sharp peaks, corresponding to siderite with a crystallite size of ∼ 8 nm with no further peaks suggesting the absence of any crystalline mineral impurities (Figure S20a). We used the same batch of siderite for all experiments and analyses and although the siderite was stored in suspension, further XRD measurements did not show any changes in crystallinity or other ageing effects.

S3 Detailed Analytical Methods

S3.1 High Performance Liquid Chromatography (HPLC)

HPLC methods used to quantify the organic ligands are provided in Table S1. For our analyses we used an Agilent 1100 series HPLC instrument, equipped with a DAD-detector, and an

Agilent ZORBAX Eclipse XDB-C18 column (4.6 mm x 150 mm, 5 µm). The mobile phase was a mixture of HPLC grade methanol and 50 mM dihydrogen phosphate buffer adjusted to pH 2.5 using HPLC grade ortho-phosphoric acid. For EDTA analysis, $FeCl_3$ solution was added to all standards and samples to a final concentration of 1 mM Fe.

Table S 1: Parameters used for HPLC analyses of the organic ligands.

**Ligand**	**Citrate**	**Salicylate**	**Tiron**	**EDTA**
**Mobile phase**	100 % phosphate buffer, pH 2.5	45 % phosphate buffer, 55% MeOH	50 % phosphate buffer, 50 % MeOH	30 % phosphate buffer, 70 % MeOH
**Flow rate**	1 ml/min	1 ml/min	1 ml/min	1 ml/min
**Temperature (°)**	20	35	50	50
**Injection volume**	8 µL	5 µL	5 µL	8 µL
**Wavelength**	214 nm	235 nm	235 nm	214 nm
**Runtime**	20 min	15 min	15 min	15 min

S3.2 Mössbauer Spectroscopy

^{57}Fe Mössbauer spectroscopy (WissEl, Wissenschaftliche Elektronik GmbH equipped with a closed-cycle He cyrostat (Janis Research SHI-850-5)) was used to determine the reduction extent and the nature of the siderite and associated oxidation products. Samples were prepared by filtering onto a membrane (0.45 μm, cellulose) inside the anaerobic chamber, which was sandwiched between two pieces of Kapton tape. Spectra were collected in transmission mode between 4 K and 140 K using a ^{57}Co source and were calibrated against α-Fe metal foil. Recoil software (University of Ottawa, Canada[4]) was used to fit the spectra using Voigt spectral lines.

Mössbauer measurements were made at 140 K and 77 K to aid with differentiation of goethite, lepidocrocite and ferrihydrite and to gain information about sample crystallinity based on their ordering temperatures [5, 6]. Select samples were measured at 4 K although due to the difficulty of fitting the siderite octet, we only fit the 4 K spectra of siderite in the absence of organic ligands after 24 hours or with EDTA, in which little or no siderite remained. The sample of siderite with EDTA after 24 hours was also measured at 295 K to allow for easier Fe(II)/Fe(III) determination in the presence of poorly ordered phases. As ferrihydrite and lepidocrocite are difficult to reliably differentiate in Mössbauer spectra measured at 140 K and 77 K, we do not differentiate between them in our discussion of the Mössbauer data with the exception of spectra measured at 4 K where they have distinct hyperfine field parameters.

S3.3 X-Ray Diffraction

Mineral identification was performed with powder X-ray diffraction (XRD, D8 Advance, Bruker) with Rietveld analysis (Topas, Bruker). The sample material was dried inside the anaerobic chamber, resuspended in ethanol, and mounted on a polished Si wafer (Sil'tronix Silicon

Technologies, France). To minimise oxidation, pure siderite samples were analysed within a plexiglass dome equipped with an anti-scatter knife edge, samples of oxidation products were measured directly on wafers to allow a longer measurement time and therefore higher quality data. Samples were measured in Bragg-Brentano geometry using Cu $K\alpha 1, 2$ radiation ($k_{\alpha 1}$ = 1.5406 Å, $k_{\alpha 2}$ = 1.54439 Å, 40 kV, and 40 mA) with a high-resolution energy dispersive 1D detector (LYNXEYE). Diffractograms were recorded from 10 - 70° 2θ with a step size of 0.02° 2θ and a 4 s acquisition time per step with the dome and a 10 s step acquisition time without the dome.

Crystallite thickness of lepidocrocite and goethite was calculated using the Topas software (Version 5, Bruker) by fitting the unit cell parameters using a Lorentzian peak function, the volume weighted size parameter (LVol-IB), and the Scherrer equation parameter (LVol-FWHM). Instrumental peak broadening was corrected for using NIST SRM 660c (LaB6) as a reference with a crystallite size of 800 nm.

S3.4 X-Ray Absorption Spectroscopy

Speciation of solid-phase Fe in samples was analyzed by bulk Fe K-edge (7112 eV) XAS at beamline 5-BM-D of the Advanced Photon Source (APS, Chicago, IL). For these analyses, samples were pressed into 1.0 cm pellets and sealed with Kapton tape. To prevent oxidation all samples were prepared in the anaerobic chamber, where they were doubly sealed in Al foil for transport and measured under anoxic conditions. X-ray absorption near edge structure (XANES) and extended X-ray absorption fine structure (EXAFS) spectra were recorded in transmission mode at ∼80 K using a $N_{2(l)}$ cooled spectroscopy stage (Linkam Scientific Instruments). The Si(111) monochromator was calibrated to the first-derivative maximum of the K-edge absorption spectrum of a metallic Fe foil (7112 eV). The foil was continuously monitored to account for small energy shifts (<1 eV) during the sample measurements. Higher harmonics in the beam were eliminated by detuning the monochromator by 40% of its maximal intensity. Two to four scans per sample were collected and averaged. All spectra were energy calibrated, pre-edge subtracted, and postedge normalized in Athena[7] with the edge energy, E_0, defined as the zero-crossing in the second XANES derivative. Linear combination fitting of the EXAFS and XANES spectra were performed as described in section S10.

S4 Organic Ligand Properties

The structures of organic ligands used in this study are provided in Figure S1 and the respective pKa values in Table S2. Ionic strength (I) was calculated for our approximate solution conditions although we did not account for dissolved Fe(II) where present.

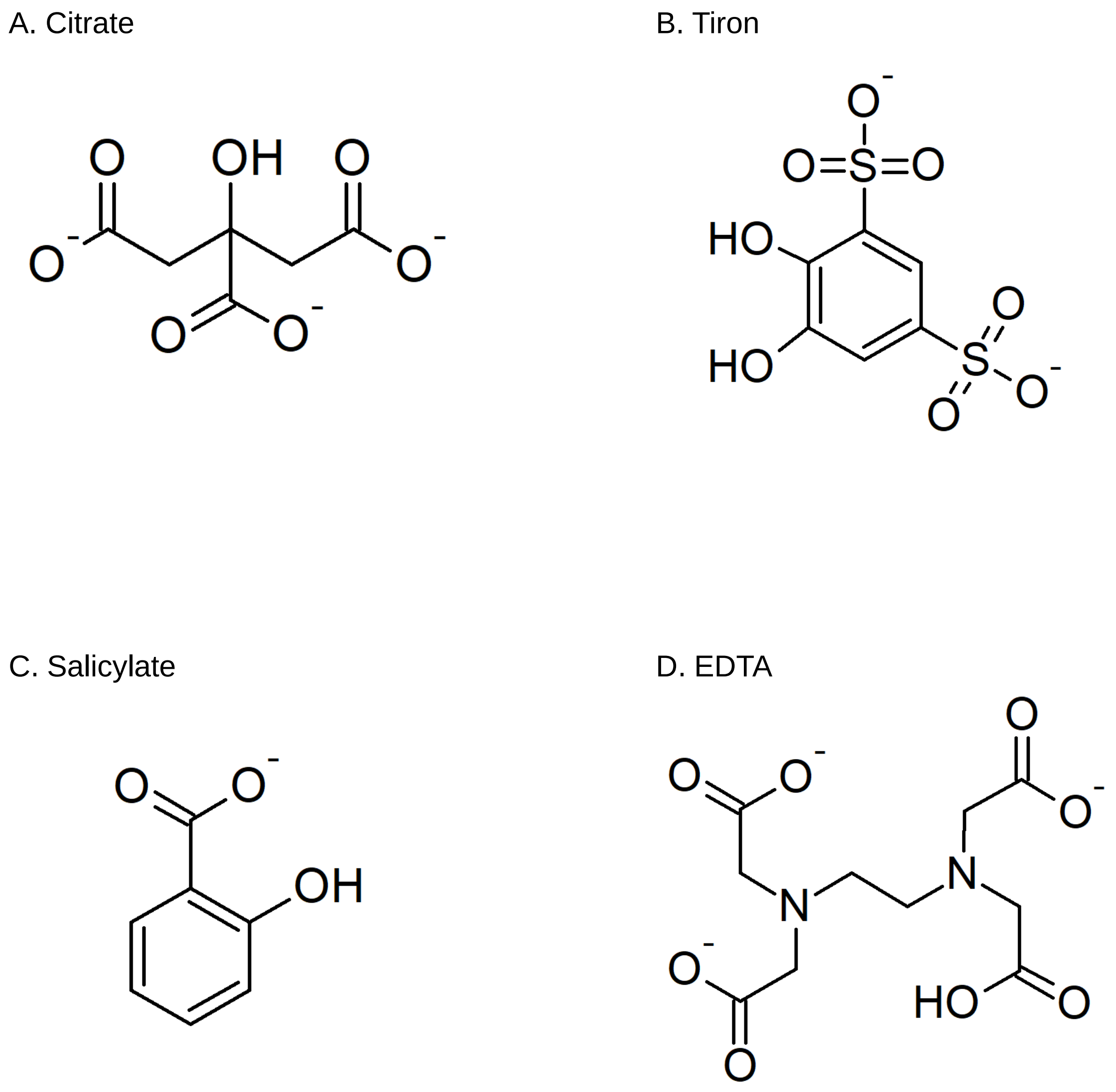

Figure S 1: Structures of the organic ligands used in this study including (a) citric acid, (b) 4,5-dihydroxy-1,3-benzenedisulfonic acid (tiron), (c) salicylic acid, and (d) Ethylenediaminetetraacetic acid (EDTA).

Table S 2: pK_a values for the acids used in this study, corrected for I using the Davies Equation. Data is taken from ref [8].

	**Citrate**	**EDTA**	**Salicylate**	**Tiron**
pKa1	3.14	0.00	2.93	-
pKa2	4.76	1.50	13.61	-
pKa3	6.40	2.00	-	7.84
pKa4	-	2.69	-	12.73
pKa5	-	6.13	-	-
pKa6	-	10.37	-	-

Table S 3: Stability constants used to model the speciation our reactors. Values are valid for 25°C and are taken from the IUPAC stability constants database [9] and adjusted for I using the Davies Equation unless otherwise specified.

**Reaction**	**log K**
**OH**	
$Fe^{2+} + H_2O - H^+ = FeOH^+$	-9.41
$Fe^{2+} + 2H_2O - 2H^+ = Fe(OH)_2{}^0$	-23.8
$Fe^{2+} + 3H_2O - 3H^+ = Fe(OH)_3^-$	-27.79
$Fe^{3+} + H_2O - H^+ = FeOH^{2+}$	-2.82
$Fe^{3+} + 2H_2O - 2H^+ = Fe(OH)_2{}^+$	-5.56
$Fe^{3+} + 3H_2O - 3H^+ = Fe(OH)_3{}^0$	-11.14
$Fe^{3+} + 4H_2O - 4H^+ = Fe(OH)_4{}^-$	-20.72
$2Fe^{3+} + 2H_2O - 2H^+ = Fe_2(OH)_2{}^{4+}$	-3.06
$2Fe^{3+} + 4H_2O - 4H^+ = Fe_3(OH)_4{}^{5+}$	-6.12
**EDTA**	
$H^+ + EDTA^{4-} = HEDTA^{3-}$	10.948
$2H^+ + EDTA^{4-} = H_2EDTA^{2-}$	17.221
$3H^+ + EDTA^{4-} = H_3EDTA^-$	20.34
$4H^+ + EDTA^{4-} = H_4EDTA^0$	22.554
$5H^+ + EDTA^{4-} = H_5EDTA^+$	24.054
$6H^+ + EDTA^{4-} = H_6EDTA^{2+}$	23.84
$Fe^{2+} + EDTA^{4-} = FeEDTA^{2-}$	16.014
$Fe^{2+} + H^+ + EDTA^{4-} = FeHEDTA^-$	19.054
$Fe^{3+} + EDTA^{4-} = FeEDTA^-$	27.671
$Fe^{3+} + H^+ + EDTA^{4-} = FeHEDTA^0$	29.186
$Fe^{3+} + EDTA^{4-} + H_2O = Fe(OH)EDTA^{2-}$	19.873
**Citrate**	
$H^+ + Cit^{3-} = HCit^{2-}$	5.76

$2H^{+} + Cit^{3-} = H_2Cit^{-}$	10.33
$3H^{+} + Cit^{3-} = HCit^{0}$	13.34
$Fe^{2+} + Cit^{3-} = FeCit^{-}$	4.97
$Fe^{2+} + Cit^{3-} + H^{+} = FeHCit^{0}$	5.96
$Fe^{2+} + Cit^{3-} + 2H^{+} = FeH_2Cit^{+}$	9.91
$Fe^{2+} + 2Cit^{3-} + H^{+} = FeHCit_2{}^{3-}$	11.99
$2Fe^{2+} + 2Cit^{3-} + 2H_2O - 2H^{+} = Fe_2(OH)_2Cit_2{}^{4-}$	-2.53
Fe2+ + $Cit^{3-} + H_2O - H^{+} = FeOHCit^{2-}$	
$Fe^{3+} + Cit^{3-} = FeCit^{0}$	11.21
$Fe^{3+} + Cit^{3-} + H^{+} = FeHCit^{+}$	12.28
$Fe^{3+} + 2Cit^{3-} + H^{+} = FeHCit_2{}^{+}$	19.12
$Fe^{3+} + Cit^{3-} + H_2O - H^{+} = FeOHCit^{-}$	8.51
$2Fe^{3+} + 2Cit^{3-} + 2H_2O - 2H^{+} = Fe_2(OH)_2Cit_2{}^{2-}$	21.2
**Tiron**	
$H^{+} + Tir^{4-} = HTir^{3-}$	12.5
$2H^{+} + Tir^{4-} = H_2Tir^{2-}$	20.12
$Fe^{2+} + Tir^{4-} = FeTir^{2-}$	8.92
$Fe^{2+} + H^{+} + Tir^{4-} = FeHTir_2{}^{-}$	15.71
$Fe^{2+} + 2Tir^{4-} = FeTir_2{}^{6-}$	15.33
$Fe^{3+} + Tir^{-} = FeTir^{-}$	20.32
$Fe^{3+} + 2Tir^{4-} = FeTir_2{}^{5-}$	36.95
$Fe^{3+} + Tir^{4-} + H^{+} = FeHTir^{0}$	22.12
$Fe^{3+} + 3Tir^{4-} = FeTir_3{}^{9-}$	46.06
**Salicylate**	
$H^{+} + Sal^{2-} = HSal^{-}$	13.66
$2H^{+} + Sal^{2-} = H_2Sal^{0}$	16.64
$Fe^{2+} + Sal^{2-} = FeSal^{0}$	6.55
$Fe^{2+} + 2Sal^{2-} = FeSal_2{}^{2-}$	11.2
$Fe^{2+} + Sal^{2-} + H^{+} = FeHSal^{+}$	4.80
$2Fe^{2+} + Sal^{2-} + H^{+} = Fe_2HSal^{3+}$	8.30
$Fe^{3+} + Sal^{2-} = FeSal^{+}$	16.45
$Fe^{3+} + 2Sal^{2-} = FeSal_2{}^{-}$	29.12
$Fe^{3+} + 3Sal^{2-} = FeSal_3{}^{3-}$	40.89
$Fe^{3+} + Sal^{2-} + H^{+} = FeHSal^{2+}$	4.40

Table S 4: Calculated E_H values for important redox couples in our reactors, corrected for I using the Davies Equation. Data is taken from ref [8].

**Redox Couple**	**E_H (V)**
Fe^{2+}/Fe^{3+}	0.73
$FeEDTA^{2-}/FeEDTA^{-}$	0.15
$FeOHEDTA^{3-}/FeOHEDTA^{2-}$	0.28
$FeCit^{-}/FeCit^{0}$	0.41
$FeOHCit^{2-}/FeOHCit^{-}$	0.23
$FeTir^{2-}/FeTir^{-}$	0.11
$FeHTir^{-}/FeHTir^{0}$	0.40
$FeSal^{0}/FeSal^{+}$	0.20

S5 Aqueous Speciation of Fe

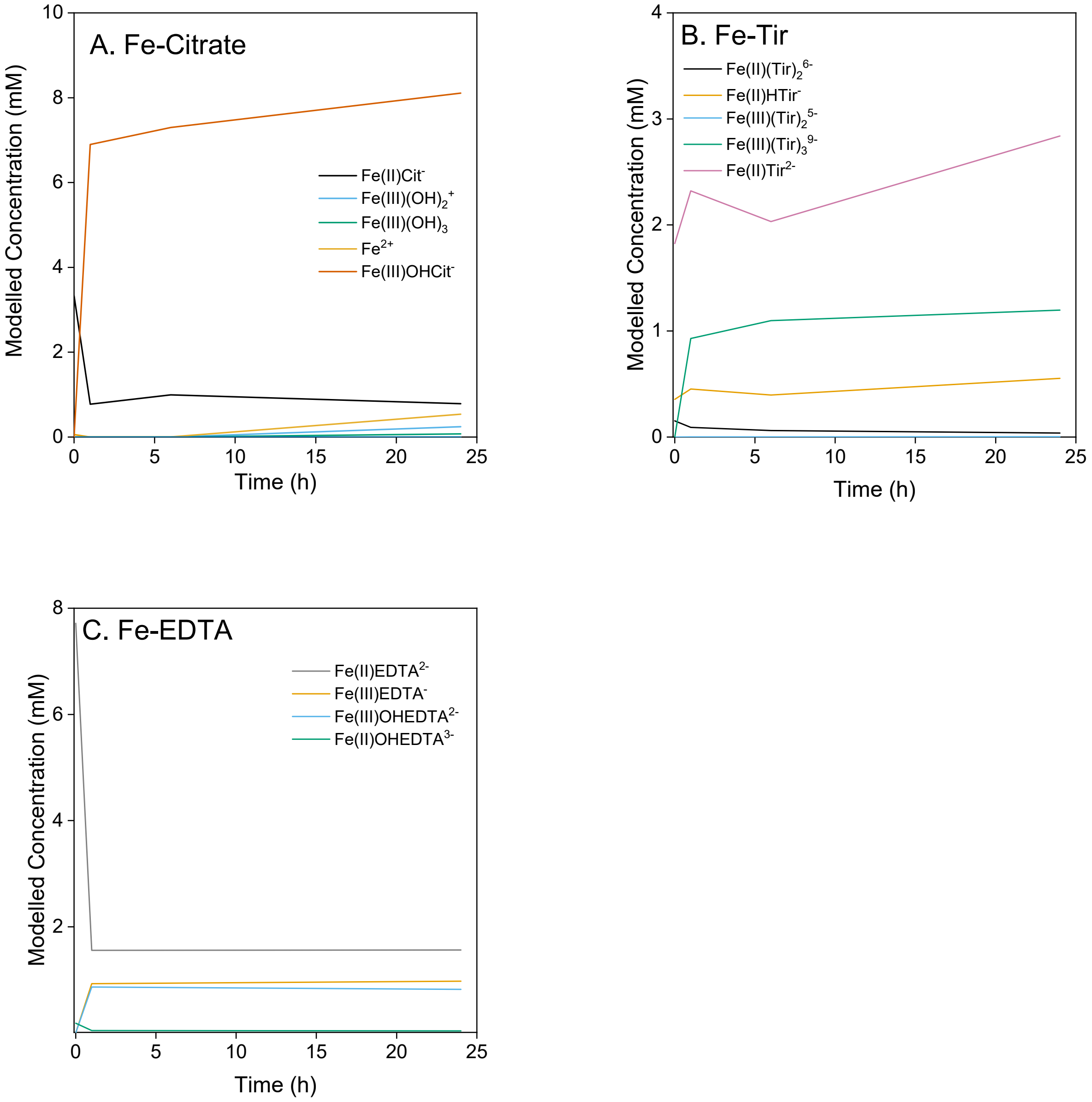

Figure S 2: Modelled aqueous Fe speciation of our reactors containing (a) citrate, (b) tiron, and (c) EDTA. The calculations were performed using PHREEQC and were based on our measured aqueous Fe and ligand concentrations. Data for salicylate is not shown as no aqueous Fe was measured. Some modelled species are not shown if they are present at very low concentrations. Data for the concentration of aqueous Fe resulting from siderite dissolution is provided in Figure S3 and Table S5.

S6 Fe Dissolution

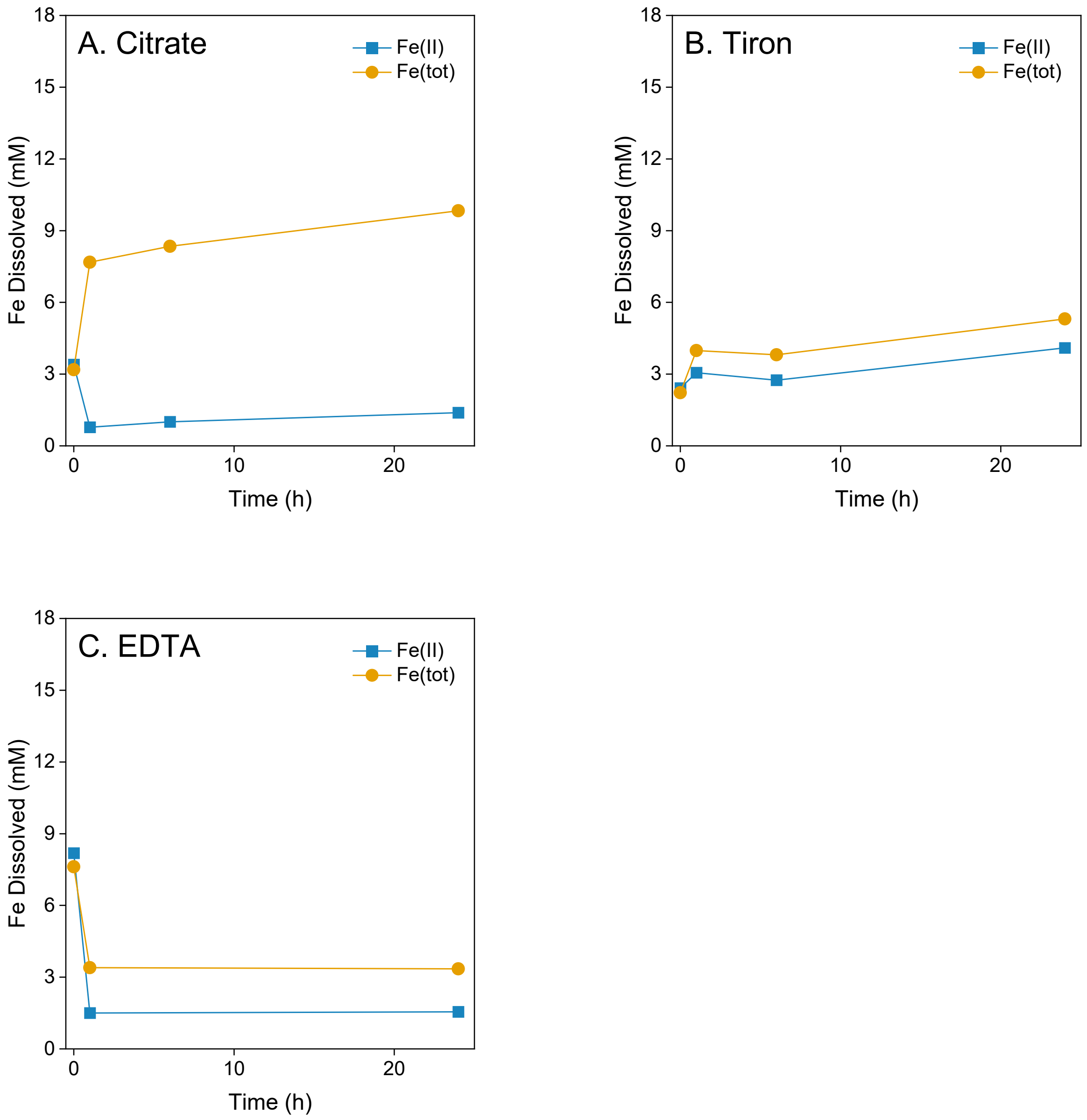

Figure S 3: Extent and Fe(II)/(tot) ratios of the aqueous Fe resulting from siderite dissolution over time in the presence of (a) citrate, (b) tiron, and (c) EDTA, over 24 hours following the onset of oxidation (Time 0). The siderite and ligands had been allowed to equilibrate for 24 hours previously inside the anaerobic chamber. Data for siderite alone in buffer, and for salicylate is not shown as no dissolution was measured. Error bars are smaller than the symbols and are not shown.

S6.1 Aqueous Fe(II)

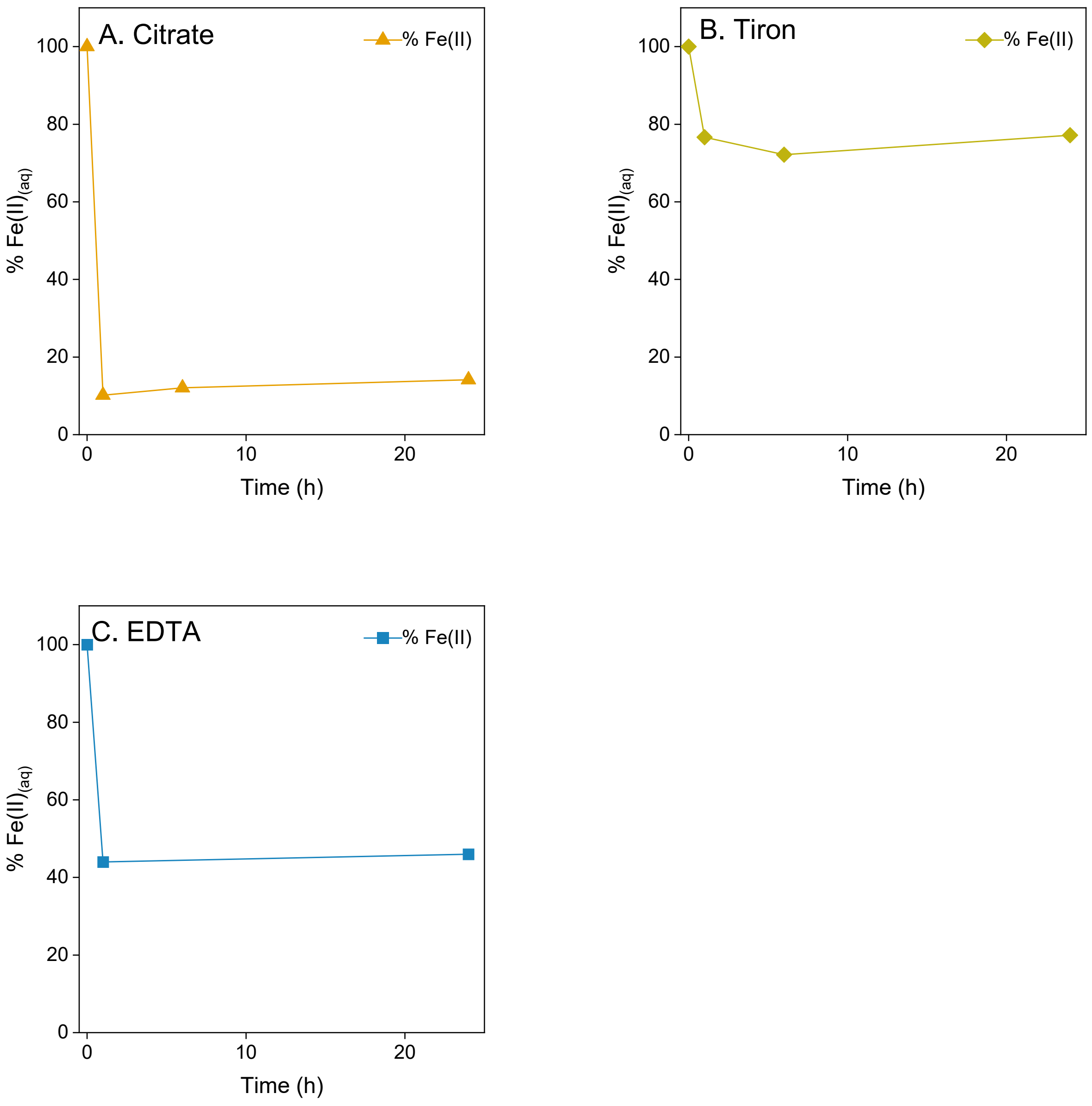

Figure S 4: Percentage Fe(II)/Fe(tot) over time of aqueous Fe(II) resulting from siderite dissolution in the presence of (a) citrate, (b) tiron, and (c) EDTA, over 24 hours following the onset of oxidation (Time 0). The siderite and ligands had been allowed to equilibrate for 24 hours previously inside the anaerobic chamber. Data for siderite alone in buffer, and for salicylate is not shown as no dissolution was measured. Error bars are smaller than the symbols and are not shown.

Table S 5: Measured dissolution of Fe from the siderite structure after 24 hours of anoxic equilibration ("Time 0") and 24 hours of stirring in an oxygen saturated suspension ("Time 24"). The aqueous Fe concentration and aqueous Fe(II)/Fe(tot) ratios were measured using the 1,10-phenanthroline method. The %Fe(II)/Fe(tot) refers to the aqueous phase only. The % total Fe dissolved refers to the percentage of Fe present in the aqueous phase relative to the remaining solid phase. Before anoxic equilibration, the total Fe was 17 mM added as siderite.

	**Time 0**			**Time 24**		
	**Fe$_{aq}$ (mM)**	**% Fe(II)$_{aq}$/Fe(tot)$_{aq}$**	**% Total Fe Dissolved**	**Fe$_{aq}$ (mM)**	**% Fe(II)$_{aq}$/Fe(tot)$_{aq}$**	**% Total Fe Dissolved**
**Siderite alone**	-	-	-	-	-	-
**Salicylate**	-	-	-	-	-	-
**Citrate**	3.2 $\pm$ 0.6	100	19	9.8 $\pm$ 0.3	14	58
**EDTA**	7.9 $\pm$ 0.4	100	46	3.4 $\pm$ 0.4	46	20
**Tiron**	2.3 $\pm$ 0.1	100	14	5.3 $\pm$ 0.4	77	31

S7 Ligand Sorption

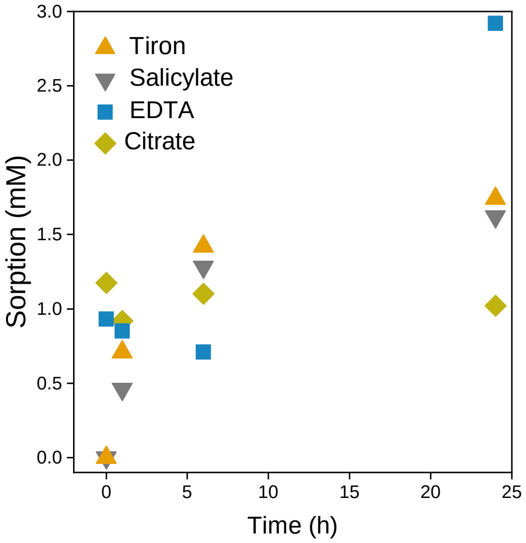

Figure S 5: Sorption of the organic ligands to the mineral surface over time, measured using HPLC. Error bars are smaller than the symbols and are not shown.

S8 Oxidation Kinetics

Table S 6: Pseudo-first order rate constants for the oxidation of siderite in the presence and absence of organic ligands. For siderite alone in buffer we only observed one reaction phase whereas for citrate, EDTA, tiron, and salicylate, we observed a rapid, initial phase (Phase 1) followed by a slower, second phase. For salicylate we could not model any portion of the reaction kinetics according to a rate law.

	**log k (h^{-1})**
**Siderite alone**	-0.14 ± 0.03
	**log k (h^{-1}), phase 1**
**Citrate**	0.11 ± 0.02
**EDTA**	-0.40 ± 0.03
**Tiron**	-0.84 ± 0.08
**Salicylate**	-

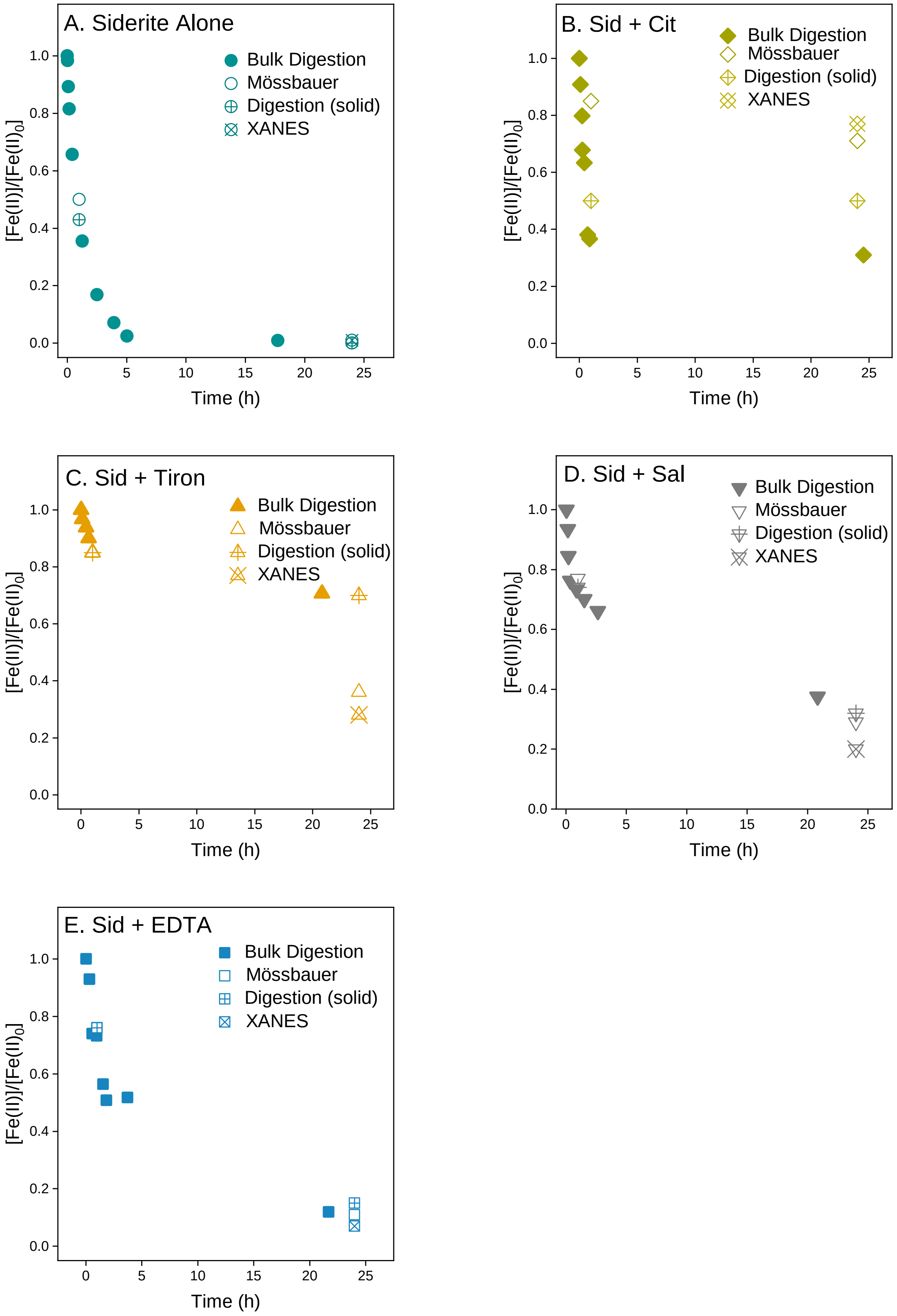

Figure S 6: Bulk Fe(II) oxidation kinetics in siderite suspensions containing (a) siderite in buffer alone, (b) citrate, (c) tiron, (d) salicylate, and (e) EDTA. Filled symbols show data measured by bulk digestion in 5 M HCl, unfilled symbols show data for the solid phase determined using Mössbauer spectroscopy, symbols filled with a "+" represent digestion of filtered solids, and symbols filled with an "X" represent the solid phase determined by XANES.

Table S 7: Comparison of the % Fe(II)/Fe(tot) ratios of the solid phase measured using the 1,10-phenanthroline method following digestion in 5 N HCl and the % Fe(II)/Fe(tot) ratios measured using Mössbauer spectroscopy after 1 hour and using Mössbauer spectroscopy (MB) and XANES after 24 hours. The % Fe(II)/Fe(tot) ratios for the digested samples have been corrected to only show the Fe content of the solid phase, adjusting for the dissolution we measured in the solid phase in the presence of citrate, tiron, and EDTA. Δ is the discrepancy between the measured solid % Fe(II)/Fe(tot) ratio according to Mössbauer spectroscopy and XANES and the bulk % Fe(II)/Fe(tot) measured through digestion.

	**% Fe(II), 1 hour**			**% Fe(II), 24 hours**			
	**MB**	**Digestion**	**Δ**	**MB**	**XANES**	**Digestion**	**Δ**
**Sid alone**	50	43	-7	0	1	0	0/-1
**Citrate**	85	50	-35	71	77	50	-21/-27
**Salicylate**	77	74	-3	29	20	32	+3/+12
**EDTA**	76	76	0	11	7	15	+4/+8
**Tiron**	85	85	0	36	28	70	+34/+42

S9 Mössbauer Spectra and Fits

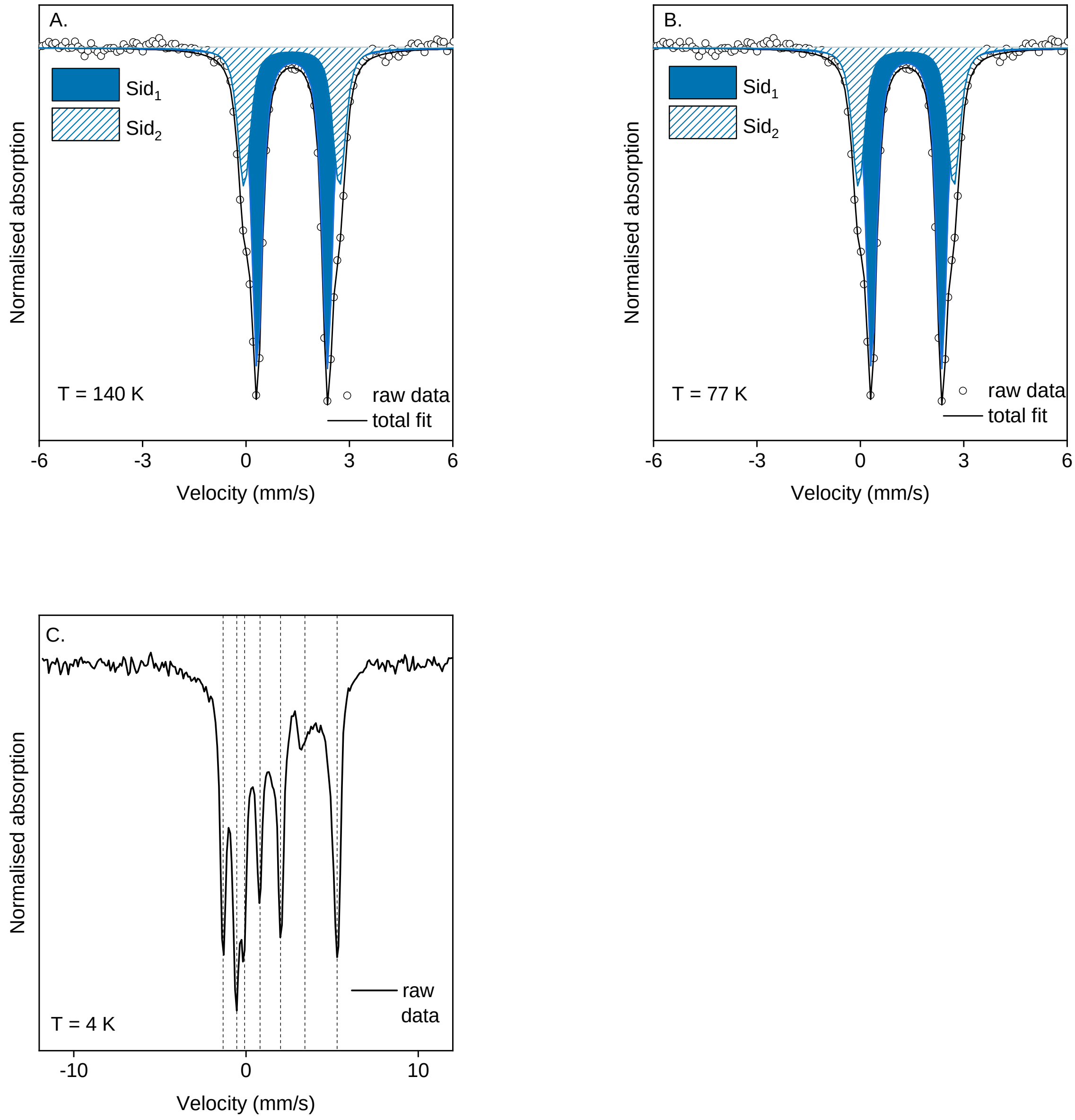

Figure S 7: Mössbauer spectra of the synthetic siderite measured at (a) 140 K, (b) 77 K, and (c) 4 K. At 4 K we were unable to satisfactorily fit the spectra however we include dashed lines to show the positions of peaks that are characteristic of magnetically ordered siderite.

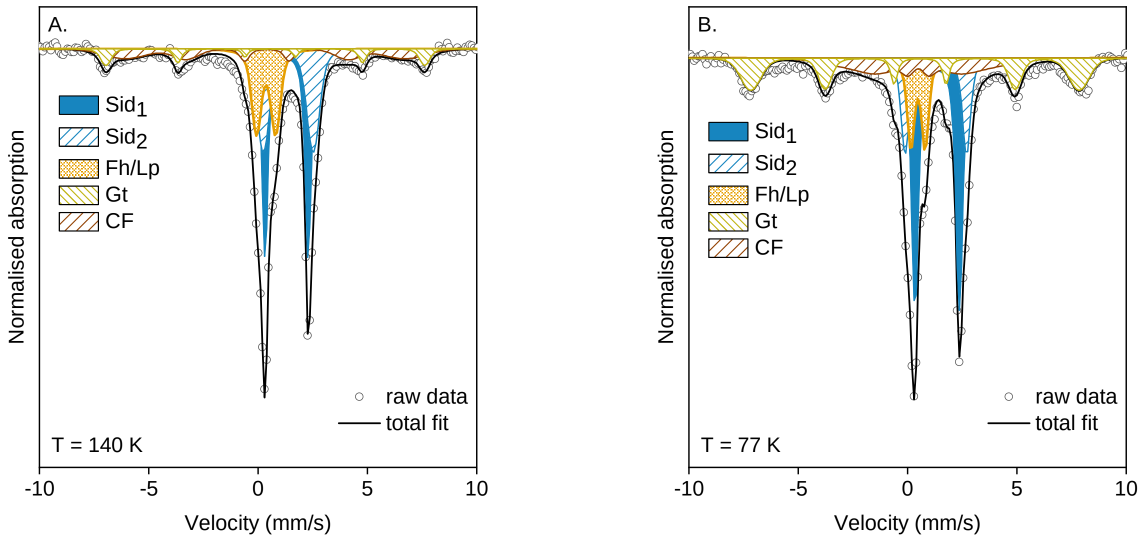

Figure S 8: Mössbauer spectra of siderite after 1 hour oxidation measured at (a) 140 K, and (b) 77 K.

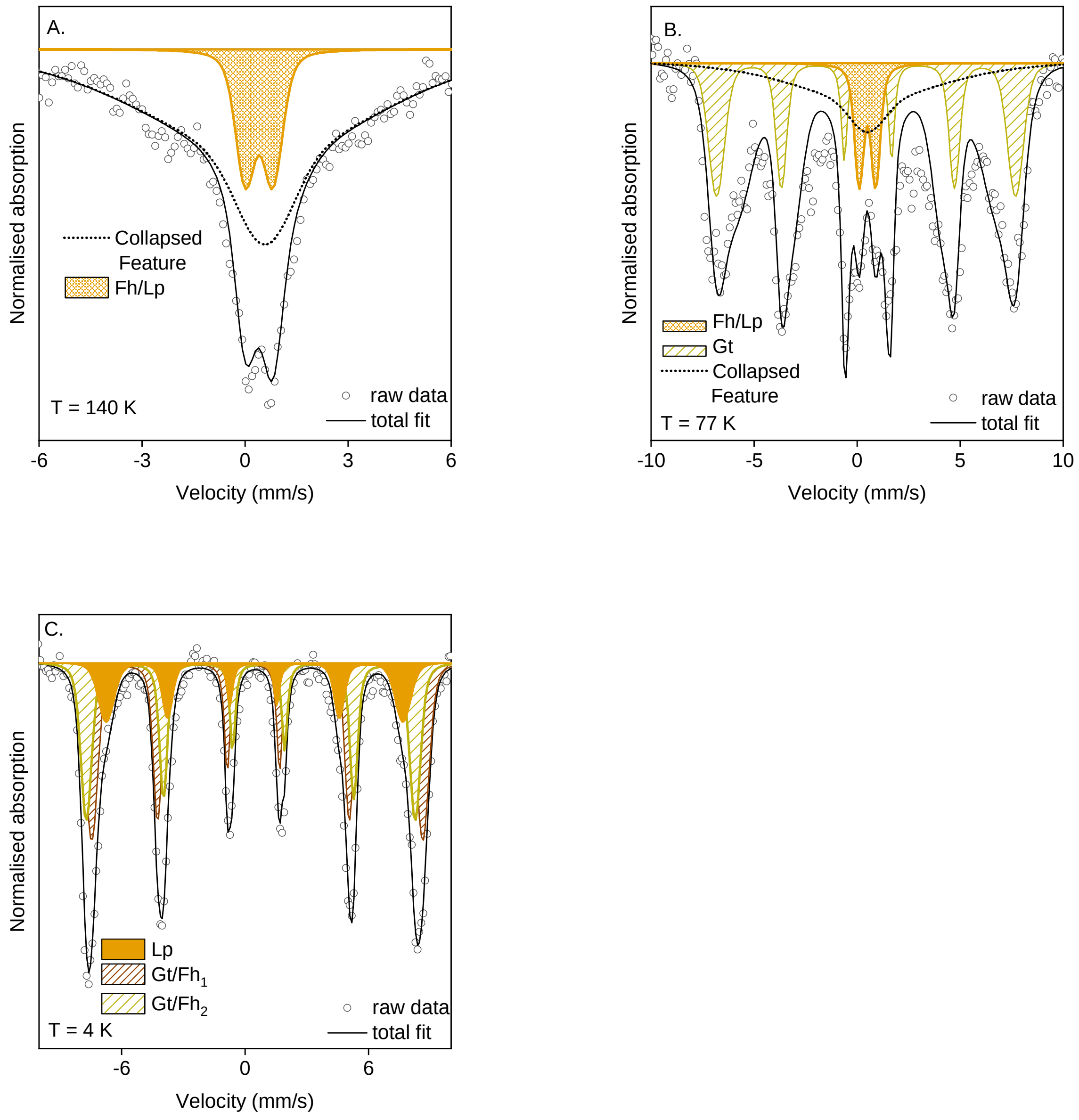

Figure S 9: Mössbauer spectra of siderite after 24 hours oxidation measured at (a) 140 K, (b) 77 K, and (c) 4 K.

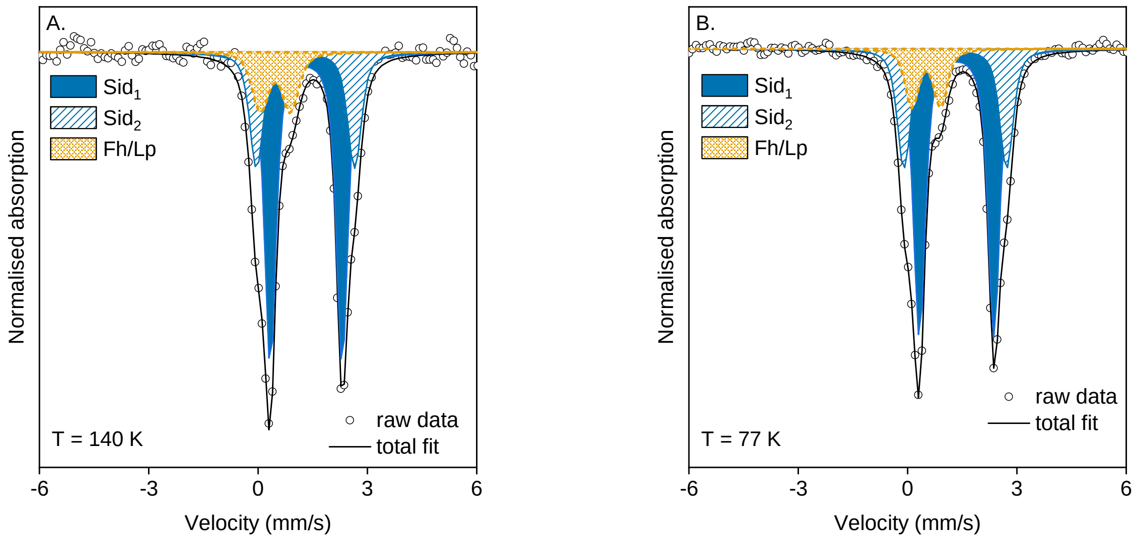

Figure S 10: Mössbauer spectra of siderite in the presence of 10 mM citrate after 1 hour oxidation measured at (a) 140 K, and (b) 77 K.

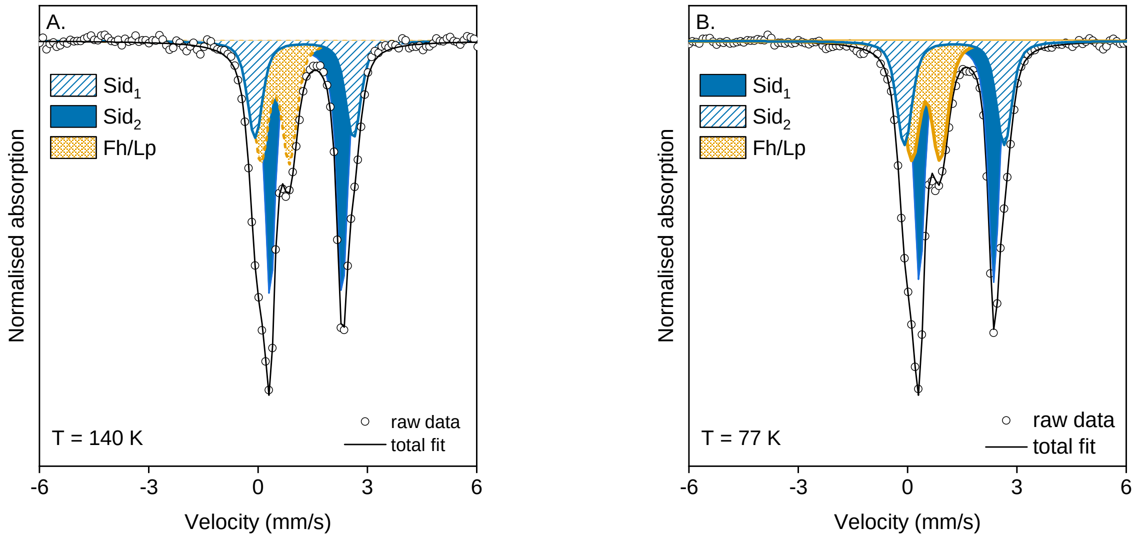

Figure S 11: Mössbauer spectra of siderite in the presence of 10 mM citrate after 24 hours oxidation measured at (a) 140 K, and (b) 77 K.

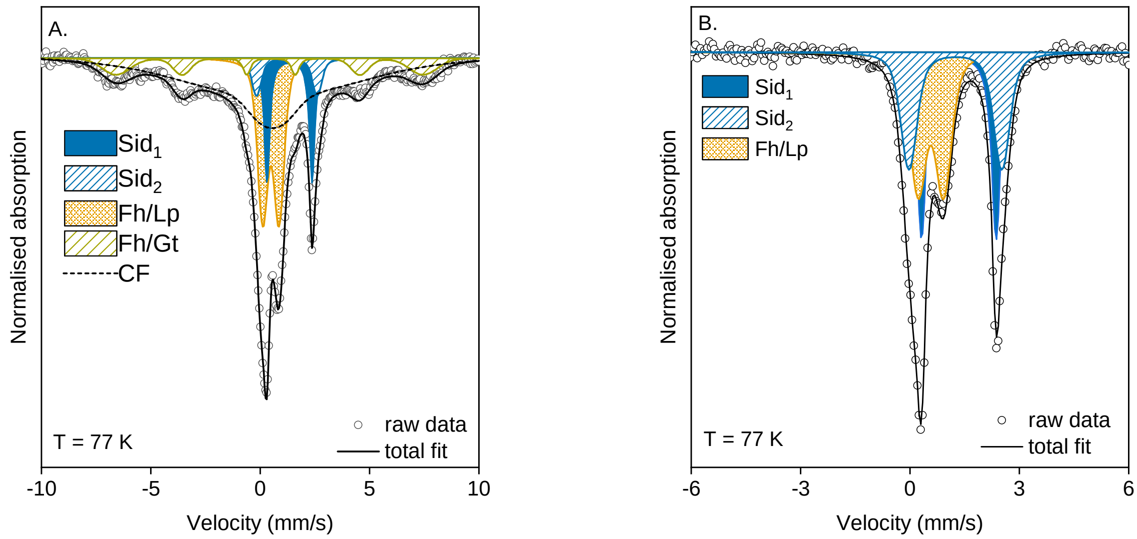

Figure S 12: Mössbauer spectra of siderite in the presence of (a) 0.1 mM citrate, and (b) 1 mM citrate after 24 hours oxidation measured at 77 K.

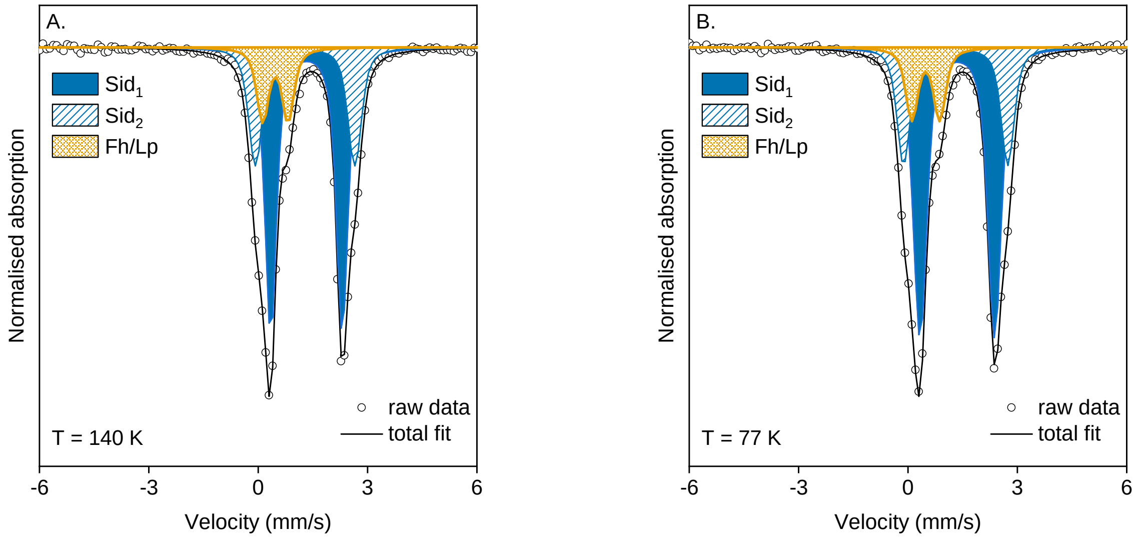

Figure S 13: Mössbauer spectra of siderite in the presence of 10 mM tiron after 1 hour oxidation measured at (a) 140 K, and (b) 77 K.

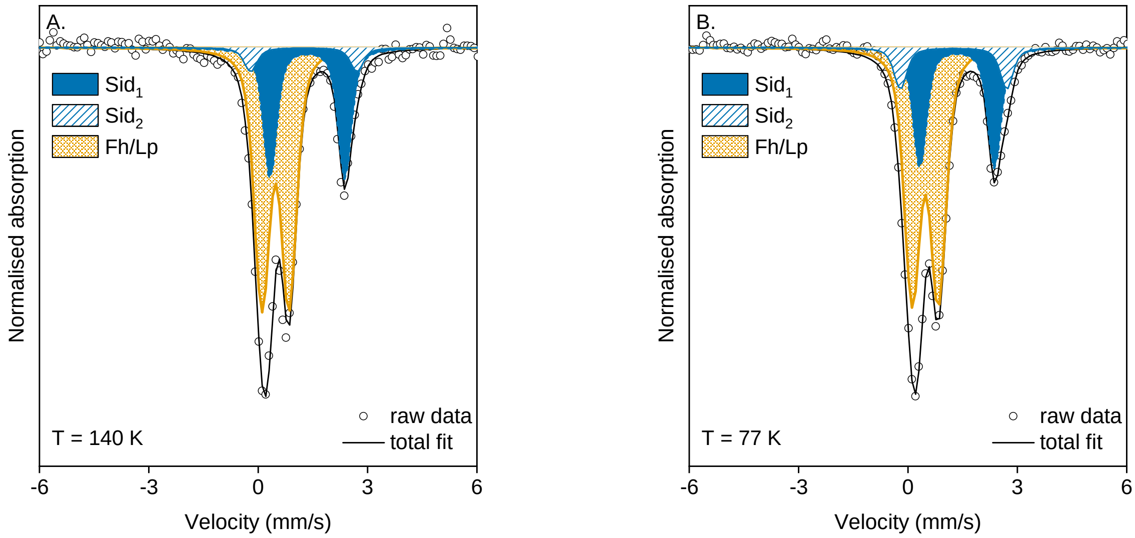

Figure S 14: Mössbauer spectra of siderite in the presence of 10 mM tiron after 24 hours oxidation measured at (a) 140 K, and (b) 77 K.

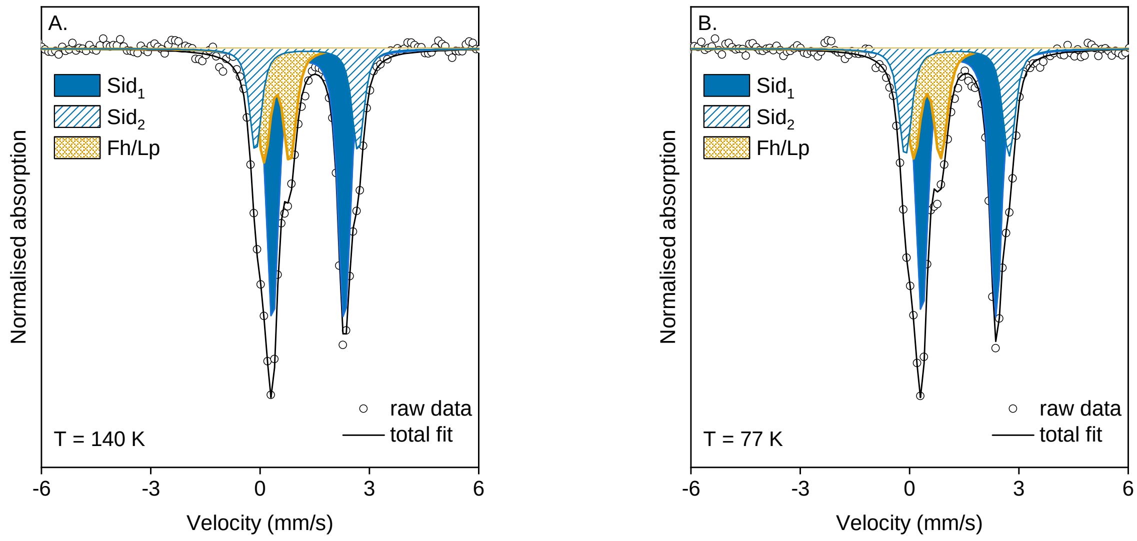

Figure S 15: Mössbauer spectra of siderite in the presence of 10 mM salicylate after 1 hour oxidation measured at (a) 140 K, and (b) 77 K.

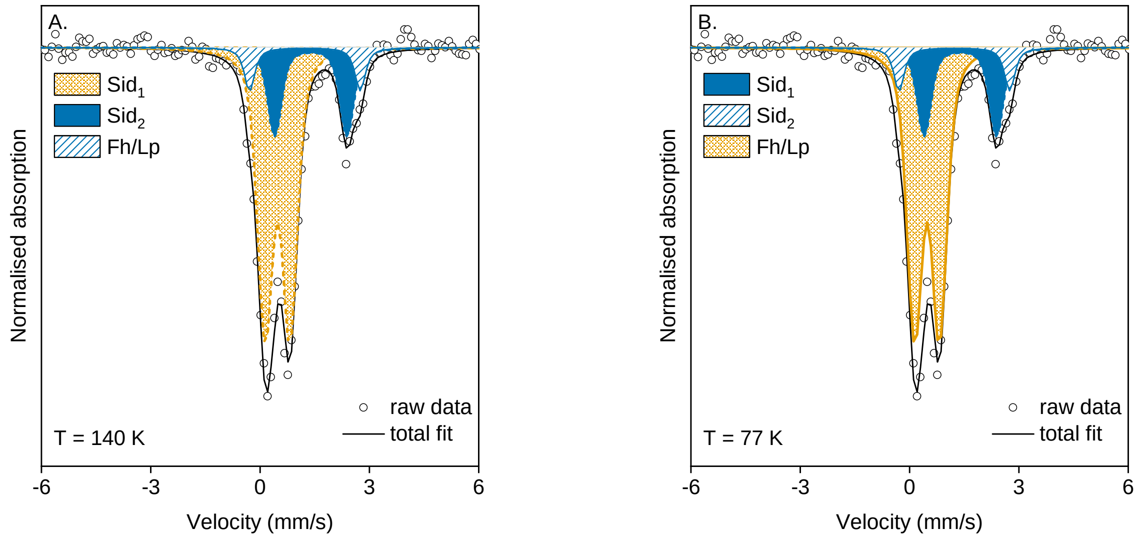

Figure S 16: Mössbauer spectra of siderite in the presence of 10 mM salicylate after 24 hours oxidation measured at (a) 140 K, and (b) 77 K.

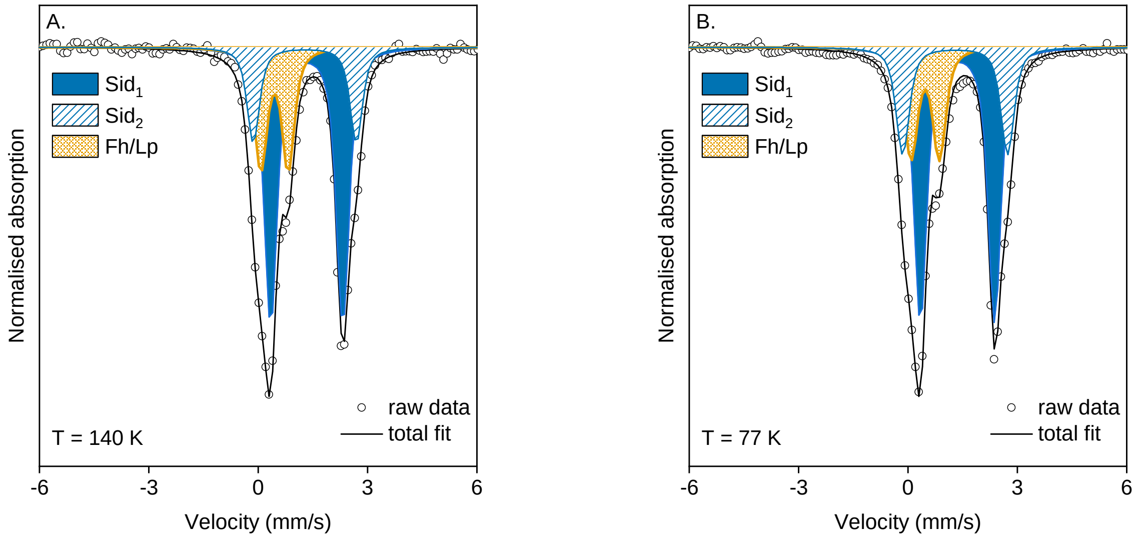

Figure S 17: Mössbauer spectra of siderite in the presence of 10 mM EDTA after 1 hour oxidation measured at (a) 140 K, and (b) 77 K.

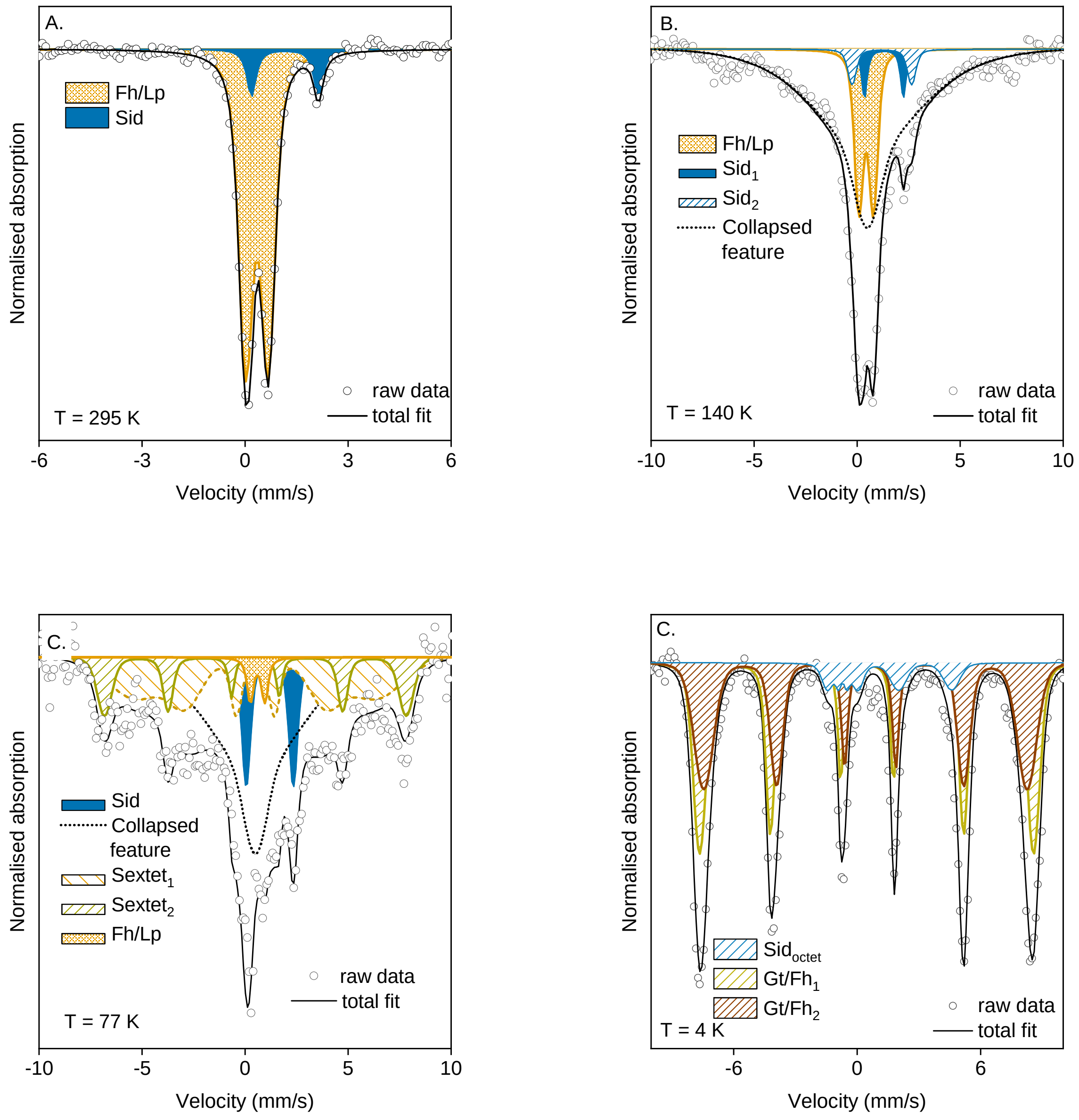

Figure S 18: Mössbauer spectra of siderite in the presence of 10 mM EDTA after 24 hours oxidation measured at (a) 295 K, (b) 140 K, (c) 77 K, and (d) 4 K.

Table S 8: Mössbauer fit parameters (VBF model).

**Sample**	**Temp (K)**	χ^{2a}	**Site**	**CS**^{b} **(mm/s)**	**QS**/ϵ^c **(mm/s)**	**H**^{d} **(T)**	σ^e **(mm/s or T)**	**Area (%)**
Pure sid	140	0.87	Sid_1	1.39	2.00		0.15	68.2
			Sid_2	1.33	2.70		0.2	31.8
Pure sid	77	0.67	Sid_1	1.39	2.04		0.17	66.9
			Sid_2	1.33	2.76		0.19	33.1
Pure sid 1 h ox	140	1.37	Sid_1	1.31	1.97		0.08	31.5
			Sid_2	1.38	2.28		0.44	27.4
			Fh/Lp	0.37	0.88		0.3	17.9
			Gt	0.44	-0.11	45.2	1.0	7.5
			CF	0.36	-0.05	39.7	5.1	15.6
Pure sid 1 h ox	77	0.88	Sid_1	1.35	2.03		0.21	35.1
			Sid_2	1.30	2.80		0.20	15.1
			Fh/Lp	0.48	0.66		0.30	12.6
			Gt	0.47	-0.12	46.8	1.7	21.5
			CF	0.5*	0*	18.6	5.0	15.7
Pure sid 24 h ox	140	0.62	Fe(III)	0.41	0.8*		0.45	13.3
			CF	0.44	-0.14	14.4	19.6	86.7
Pure sid 24 h ox	77	0.96	Fe(III)	0.50	0.66		0.3	6.55
			Gt	0.48	-0.04	43.6	2.0	56.3
			CF	0.52	0*	26.8	41.0	37.3
Pure sid 24 h ox	4	0.88	Gt/Fh_1	0.47	-0.19	49.5	1.36	36.0
			Gt/Fh_2	0.49	0.09	49.8	0.01	45.4
			Lp	0.43	0.03*	44.7	3.1	18.6
Sid + Cit 1 h ox	140	0.61	Sid_1	1.32	1.97		0.15	59.5
			Sid_2	1.30	2.69		0.23	25.6
			Fh/Lp	0.48	0.83		0.33	15.0
Sid + Cit 1 h ox	77	0.53	Sid_1	1.34	2.05		0.16	59.2
			Sid_2	1.31	2.81		0.22	27.5
			Fh/Lp	0.51	0.79		0.23	13.3
Sid + Cit 24 h ox	140	0.57	Sid_1	1.32	1.98		0.12	48.2
			Sid_2	1.26	2.70		0.28	23.5
			Fh/Lp	0.47	0.79		0.27	28.3
Sid + Cit 24 h ox	77	0.91	Sid_1	1.34	2.04		0.13	45.1
			Sid_2	1.28	2.74		0.28	25.6
			Fh/Lp	0.50	0.79		0.31	29.2
Sid + 0.1 mM Cit	77	0.72	Sid_1	1.34	2.06		0.01	11.5
			Sid_2	1.22	2.77		0.22	5.2

24 h ox			Fh/Lp	0.49	0.74		0.33	23.0
			Fh/Gt	0.47	-0.03	43.5	4.2	11.4
			CF	0.55	0*	20.6	18.7	48.9
Siderite + 1 mM	77	0.60	Sid_1	1.34	2.05		0.05	32.7
Cit			Sid_2	1.25	2.55		0.23	31.0
24 h ox			Fh/Lp	0.57	0.70		0.31	36.4
Sid + Tir	140	0.84	Sid_1	1.32	1.96		0.16	57.5
1 h ox			Sid_2	1.29	2.73		0.23	27.4
			Fh/Lp	0.47	0.70		0.18	15.10
Sid + Tir	77	1.00	Sid_1	1.35	2.05		0.22	61.2
1 h ox			Sid_2	1.31	2.86		0.22	24.8
			Fh/Lp	0.49	0.77		0.19	14.03
Sid + Tir	140	0.47	Sid_1	1.33	2.02		0.21	30.3
24 h ox			Sid_2	1.20	3.03		0.21	5.2
			Fh/Lp	0.45	0.73		0.28	64.5
Sid + Tir	77	0.63	Sid_1	1.35	2.05		0.21	27.1
24 h ox			Sid_2	1.25	2.92		0.21	9.3
			Fh/Lp	0.48	0.72		0.3	63.6
Sid + Sal	140	0.62	Sid_1	1.33	1.97		0.18	56.1
1 h ox			Sid_2	1.28	2.81		0.17	20.9
			Fh/Lp	0.46	0.72		0.20	23.0
Sid + Sal	77	0.81	Sid_1	1.35	2.03		0.18	54.2
1 h ox			Sid_2	1.30	2.85		0.20	22.7
			Fh/Lp	0.48	0.74		0.22	23.0
Sid + Sal	140	0.62	Sid_1	1.50	1.65		0.17	6.6
24 h ox			Sid_2	1.22	2.58		0.19	23.1
			Fh/Lp	0.51	0.61		0.22	70.3
Sid + Sal	77	0.72	Sid_1	1.39	1.98		0.25	20.9
24 h ox			Sid_2	1.22	3.04		0.11	8.3
			Fh/Lp	0.48	0.68		0.28	70.8
Sid + EDTA	140	0.90	Sid_1	1.33	1.97		0.18	56.5
1 h ox			Sid_2	1.28	2.84		0.20	19.3
			Fh/Lp	0.45	0.76		0.22	24.3
Sid + EDTA	77	1.89	Sid_1	1.36	2.03		0.22	54.9
1 h ox			Sid_2	1.29	2.88		0.17	22.5
			Fh/Lp	0.47	0.78		0.23	22.65
Sid + EDTA	295	0.60	Sid_1	1.13	1.93		0.01	4.5
24 h ox			Sid_2	0.91	2.60		0.30	6.8
			Fe(III)	0.36	0.63		0.27	88.7
Sid + EDTA	140	0.91	Sid_1	1.36	2.01		0.30	10.2

24 h ox			Fh/Lp	0.41	0.87		0.40	26.1
			CF	0.50	0*	10.2	20	63.8
Sid + EDTA	77	0.64	Sid_1	1.36	2.09		0.20	7.3
24 h ox			Fe(III)	0.47	0.93		0.22 6.2	
			Gt	0.55	-0.068	44.6	3.1	20.7
			CF	0.50	0*	22.6	20	65.8
Sid + EDTA	4	1.86	Sid "Octet"[f]	1.3	2*	8*	10	8.0
24 h ox			Gt/Fh_1	0.47	-0.01	50.3	4.1	47.2
			Gt/Fh_1	0.52	-0.12	48.7	6.5	44.8

a Red-χ^2, goodness of fit;

b Centre shift;

c Quadrupole splitting (QS, for doublets) or quadrupole shift (ϵ, for sextets);

d Hyperfine field;

e Standard deviation of QS (doublets) or H (sextets);

f The fit of the octet is approximated using the parameters listed.

* Indicates values that were fied during the fitting process.

Abbreviations: Temp = temperature, sid = siderite, cit = citrate, tir = tiron, sal = salicylate, ox = oxidation, Gt = goethite, Fh = ferrihydrite, Lp = lepidocrocite, CF = collapsed feature.

S10 X-Ray Absorption Spectroscopy LCF-fitting

S10.1 XANES

To determine spectral contributions from Fe(II) and Fe(III), linear combination fit (LCF) analyses were performed over the energy range -20 to 30 eV (E - E_0) with no constraints. Initial fit fractions (99 ± 1%) were recalculated to a component sum of 100%.

Results obtained from LCF analyses of Fe K-edge XANES spectra are presented in Table S12.

Table S 9: Results from linear combination fit analyses of Fe K-edge XANES spectra.[a]

**Sample Name**	**Fe(II)**	**Fe(III)** (%)	**Initial fit fractions**	**NSSR**[b]	χ^{2c} (-)
Sid_only	1	99	101	0.03	0.0033
Cit_24hrs	77	23	100	0.02	0.0002
EDTA_24hrs	7	93	99	0.05	0.0053
Sal_24hrs	20	80	98	0.03	0.0003
Tir_24hrs	28	72	98	0.11	0.0003

[a] Initial fit fractions (99±1%) were recalculated to a sum of 100.

[b] Normalized sum of squared residuals ($100\Sigma_i$(data$_i$-fit$_i$)^2/$\Sigma_i$data^2).

[c] Fit accuracy; reduced χ^2 =($N_{idp}/N_{pts})\Sigma_i$((data$_i$-fit$_i$)/ϵ_i)^2(N_{idp}-N_{var})-1. N_{idp}, N_{pts} and N_{var} are, respectively, the number of independent points in the model fit, the total number of data points (89), and the number of variables in the fit (2). ϵ_i is the uncertainty of the ith data point.

Abbreviations: Sid_only = siderite alone after 24 hours oxidation, Cit = citrate, Sal = salicylate, Tir = tiron.

S10.2 EXAFS

In order to evaluate suitable references for LCF of Fe K-edge EXAFS spectra of siderite oxidation products, we employed principal component analysis and target transform testing (PCA-TT) using SixPack.[10] The results of the PCA analysis performed on k^3-weighted EXAFS spectra over 2-12 $Å^{-1}$ are presented for the first six components in Table S9. For these analysis, E_0 was defined as 7128 eV for samples and references. Based on the IND function, PCA of Fe K-edge EXAFS spectra indicated three statistically significant spectral components were required to suitable fit the samples, accounting for 88 % of spectral variance.

Table S 10: PCA Output Parameters.

Component	Eigen	Var.	Cum Var.	IND
1	56.62	0.399	0.399	0.95956
2	49.254	0.347	0.746	0.66392
3	16.604	0.131	0.878	0.65817
4	7.389	0.052	0.93	1.2582
5	5.849	0.041	0.971	4.0544
6	4.054	0.028	1	NA

The relevance of specific reference spectra for EXAFS LCF analyses was determined by target-transform testing of k^3-weighted Fe (k-range = 2-12 $Å^{-1}$) spectra. The quality of the transformation was evaluated by the empirical SPOIL value:[11] 0-1.5 excellent, 1.5-3 good, 3-4.5 fair, 4.5-6 acceptable, and >6 for an unacceptable reference spectrum. From all Fe references tested, the lowest SPOIL values (<6.0) were assigned to ferrihydrite, goethite, lepidocrocite, siderite. Poor spectral reconstructions with high SPOIL values (>6) were found for all other Fe species (Table S10). Because the obtained number of Fe references suitable for LCF analysis based on TT analysis was larger than the number of PCA components, all 5 Fe references were initially considered in LCF analyses. In no case did the number of fit references employed in the fits exceed the number of PCA components showing clear EXAFS features.

Table S 11: Results from Fe target-transform testing.

Reference	χ^2	NSSR[b]	SPOIL
Ferrihydrite[a]	69.4	0.1	2.2
Goethite[a]	449.8	0.3	5.1
Lepidocrocite[a]	290.5	0.1	2.3
Magnetite[a]	1674.3	0.6	11.6
Siderite[a]	229.5	0.1	2.8
Chloride Green rust[c]	1007.2	1.0	37.6
CO3 Green rust[d]	1373.4	0.9	19.1
SO4 Green rust[d]	2658.3	0.95	39.4
Hematite	3048.6	0.7	8.5
Fe(II)gluconate[a]	461.4	0.5	7.8
Fe(III)citrate[a]	618.6	0.4	6.2
Fe(III)EDTA[a]	381.8	0.5	8.6
Fe(III)oxalate	662.0	0.5	9.7
Fe2(II)oxalate	1282.2	0.7	12.2
$FeCl_3$_$H_2$0_0.1M	764.0	0.4	7.7
$FeCl_2$_$H_2$0_0.1M	511.9	0.5	7.9

[a] From ref [12]

[b] Normalized sum of squared residuals ($100\Sigma_i(\text{data}_i\text{-fit}_i)^2/\Sigma_i\text{data}^2$).

[c] References courtesy of T. Borch (Colorado State University)

[d] From ref [13]

Linear combination fit (LCF) analyses of k^3-weighted EXAFS spectra were performed in Athena[7] over a k-range of 2-12 Å^{-1} or 2-10 Å^{-1} (for Tiron and EDTA after 24 hours) with the E_0 of all spectra and reference compounds set to 7128 eV. The number of components included in the fit was successively increased and each additional component was retained in the fit when the NSSR value decreased by at least 10%.[14] No constraints were imposed on the fits, and initial fit fractions (87 ± 13%) were recalculated to a component sum of 100%.

Results of the LCF analysis of the EXAFS spectra are presented in Table S11.

Table S 12: Results from linear combination fit analyses of Fe K-edge EXAFS spectra.[a]

**Sample Name**	**Sid**	**Lp**	**Gt**	**Fh**	**Initial fit fraction** (%)	**NSSR**[b]	**red.** χ^{2c} (-)
Sid_only	5	16	15	65	98	2.8	0.154
Cit_24hrs	75	-	-	25	103	8.8	0.481
EDTA_24hrs	-	-	-	100	76	11.1	0.455
Sal_24hrs	25	75	-	-	80	11.4	0.650
Tir_24hrs	34	17	-	49	76	10.0	0.248

[a] Initial fit fractions (87±13%) were recalculated to a sum of 100.

[b] Normalized sum of squared residuals ($100\Sigma_i(\text{data}_i\text{-fit}_i)^2/\Sigma_i\text{data}^2$).

[c] Fit accuracy; reduced $\chi^2 = (N_{idp}/N_{pts})\Sigma_i((\text{data}_i\text{-fit}_i)/\epsilon_i)^2(N_{idp}\text{-}N_{var})\text{-1}$. N_{idp}, N_{pts} and N_{var} are, respectively, the number of independent points in the model fit, the total number of data points (201), and the number of variables in the fit (1-4). ϵ_i is the uncertainty of the *i*th data point.

Abbreviations: Sid_only = siderite alone after 24 hours oxidation, Cit = citrate, Sal = salicylate, Tir = tiron.

S11 X-Ray Diffraction Results

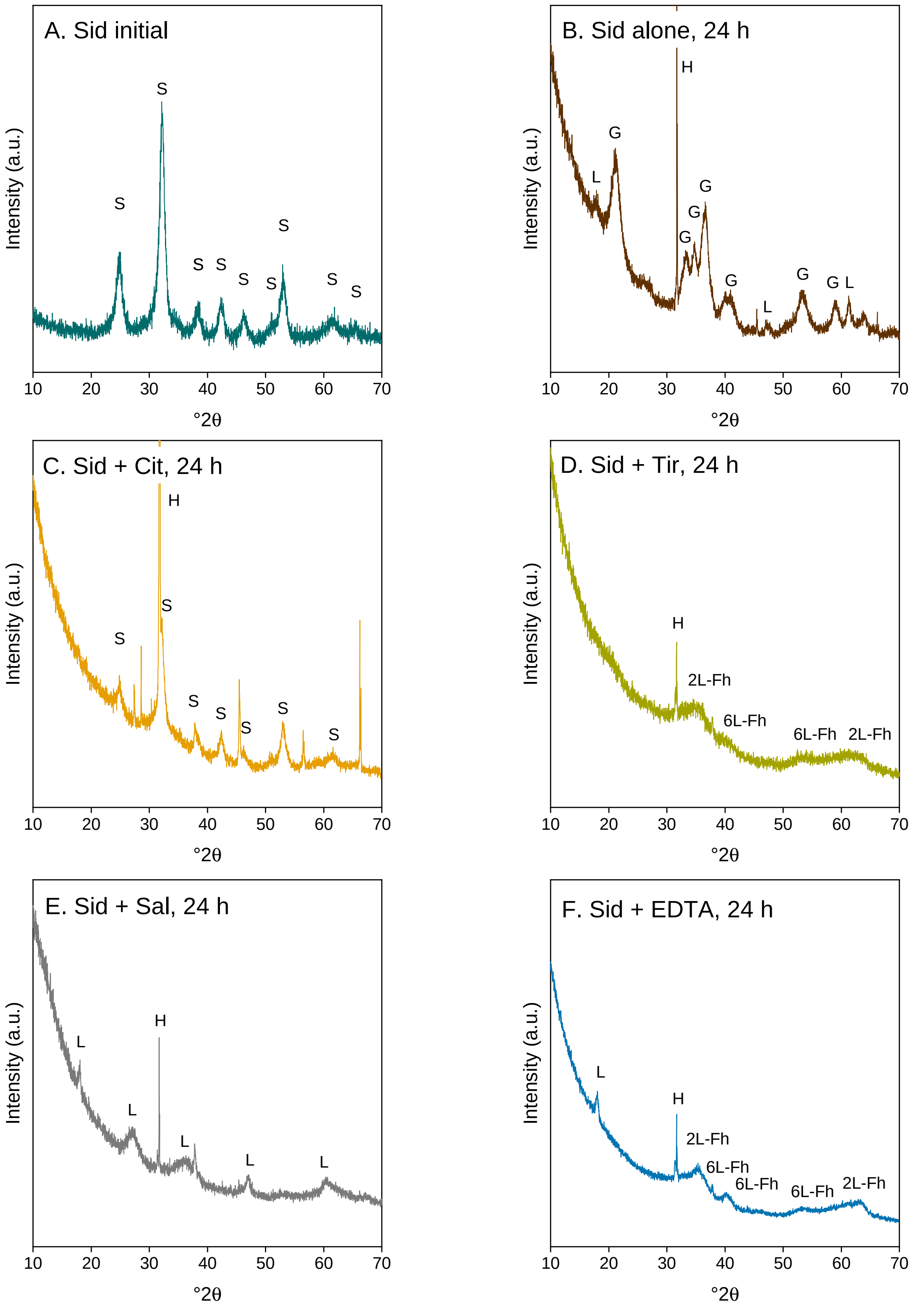

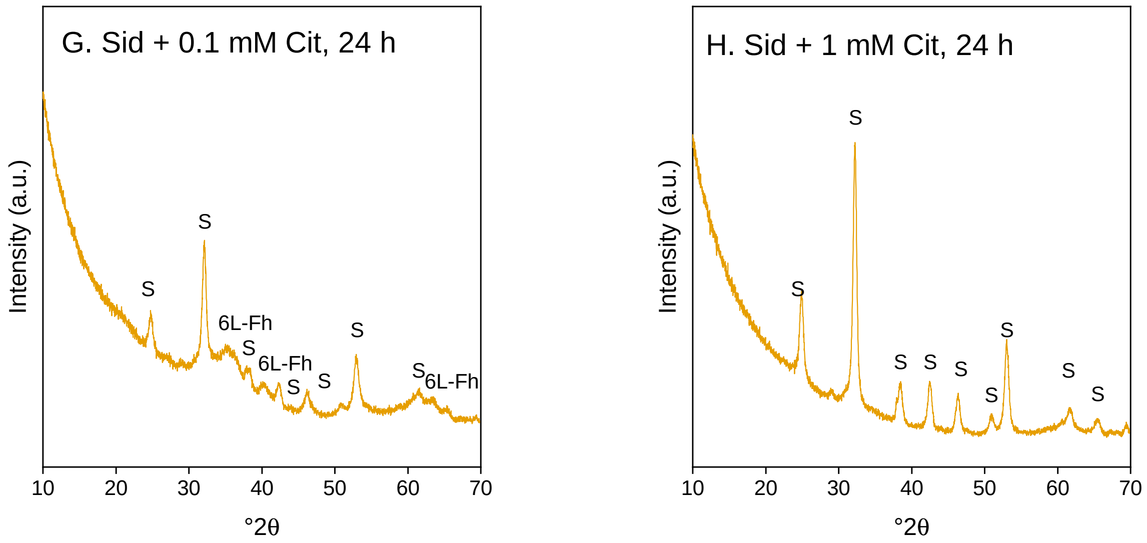

Figure S 19: XRD diffractograms of (a) pure synthetic siderite, (b) siderite alone after 24 h oxidation, (c) siderite + citrate after 24 h oxidation, (d) siderite + tiron after 24 h oxidation, (e) siderite + salicylate, and (f) siderite + EDTA after 24 h oxidation, (g) siderite + 0.1 mM citrate after 24 h oxidation, and (h) siderite + 1 mM citrate after 24 h oxidation. Diffractograms were compared against the PDF2 database. Identified peaks are labelled as 'S' for siderite, 'L' for lepidocrocite, 'G' for goethite, '2L-Fh' for 2 line ferrihydrite, '6L-Fh' for 6 line ferrihydrite, and 'H' for halite. Abbreviations: cit = citrate, tir = tiron, sal = salicylate. In order to allows comparison of the minerals of interest, the full intensity halite peak in panels (b) and (c) is not shown.

References

[1] Alfred A Schilt. *Analytical applications of 1, 10-phenanthroline and related compounds: international series of monographs in analytical chemistry.* Elsevier, 2013.

[2] Hiroki Tamura, Katsumi Goto, Takao Yotsuyanagi, and Masaichi Nagayama. Spectrophotometric determination of iron(ii) with 1, 10-phenanthroline in the presence of large amounts of iron(iii). *Talanta*, 21(4):314–318, 1974.

[3] Sudipta Rakshit, Christopher J Matocha, and Mark S Coyne. Nitrite reduction by siderite. *Soil Science Society of America Journal*, 72(4):1070–1077, 2008.

[4] Denis G Rancourt and J Y Ping. Voigt-based methods for arbitrary-shape static hyperfine parameter distributions in Mössbauer spectroscopy. *Nuclear Instruments and Methods in Physics Research Section B: Beam Interactions with Materials and Atoms*, 58(1):85–97, 1991.

[5] Enver Murad. Mössbauer spectroscopy of clays, soils and their mineral constituents. *Clay Minerals*, 45(4):413–430, 2010.

[6] Steven J Hall, Asmeret A Berhe, and Aaron Thompson. Order from disorder: Do soil organic matter composition and turnover co-vary with iron phase crystallinity? *Biogeochemistry*, 140(1):93–110, 2018.

[7] Bruce Ravel and Mathena Newville. Athena, Artemis, Hephaestus: Data analysis for X-Ray Absorption Spectroscopy using IFEFFIT. *Journal of Synchrotron Radiation*, 12(4): 537–541, 2005.

[8] Arthur E Martell, Robert M Smith, and R J Motekaitis. NIST Standard Reference Database 46. *Critical Stability Constants of Metal Complexes Database Version*, 8, 2004.

[9] Lesley D Pettit and Kipton J Powell. IUPAC Stability Constants Database. *Academic Software, Otley*, 1997.

[10] Samuel M Webb. SIXPack: A graphical user interface for XAS analysis using IFEFFIT. *Physica Scripta*, 2005(T115):1011, 2005.

[11] Edmund R Malinowski. Determination of rank by median absolute deviation (drmad): A simple method for determining the number of principal factors responsible for a data matrix. *Journal of Chemometrics: A Journal of the Chemometrics Society*, 23(1):1–6, 2009.

[12] Peggy Langner, Christian Mikutta, and Ruben Kretzschmar. Arsenic sequestration by organic sulphur in peat. *Nature Geoscience*, 5(1):66–73, 2012.

[13] Case M Van Genuchten, Thilo Behrends, Peter Kraal, Susan L S Stipp, and Knud Dideriksen. Controls on the formation of Fe (II,III)(hydr)oxides by Fe(0) electrolysis. *Electrochimica Acta*, 286:324–338, 2018.

[14] S D Kelly, D Hesterberg, and B Ravel. Analysis of soils and minerals using X-Ray Absorption Spectroscopy. *Methods of Soil Analysis. Part 5. Mineralogical Methods*, 5:387–464, 2008.
